# Clinical heterogeneity and imaging-driven genetic screening priorities in patients with radiologically suspected primary bilateral macronodular adrenal hyperplasia

**DOI:** 10.1530/EC-25-0290

**Published:** 2025-10-22

**Authors:** Huaijin Xu, Bing Li, Kang Chen, Huixin Zhou, Wangtian Ma, Yajing Wang, Yaqi Yin, Weijun Gu, Yiming Mu, Zhaohui Lyu

**Affiliations:** ^1^ School of Medicine, Nankai University, Tianjin, China; ^2^ Department of Endocrinology, The First Medical Center of Chinese PLA General Hospital, Beijing, China

**Keywords:** bilateral macronodular adrenocortical disease, bilateral adrenal macronodules, imaging feature, comorbidity, *ARMC5*

## Abstract

**Objective:**

To investigate the clinical spectrum, *ARMC5* mutation distribution, and metabolic/cardiovascular risks in patients with radiologically suspected primary bilateral macronodular adrenal hyperplasia (PBMAH).

**Design:**

Cross-sectional study.

**Methods:**

We analyzed clinical characteristics and germline *ARMC5* mutations in patients meeting radiologic criteria for PBMAH (bilateral adrenal nodules ≥1 cm), excluding non-adrenocortical lesions or bilateral adenomas with adrenal atrophy.

**Results:**

The subgroup distribution among 485 patients with radiologically suspected PBMAH was as follows: nonfunctional adrenal tumors (NFAT, 30.1%), mild autonomous cortisol secretion (MACS, 41%), overt Cushing’s syndrome (CS, 14.4%), primary aldosteronism (PA, 8.9%), and coexisting PA and MACS (PA + MACS, 5.6%). Imaging revealed a higher proportion of multiple confluent adrenal nodules in the MACS and CS groups compared to others (*P* < 0.05). Cortisol-related comorbidities (hypertension and diabetes) showed no statistically significant differences between MACS and NFAT. Germline *ARMC5* testing in 62 unrelated patients identified seven novel pathogenic variants. Pathogenic mutations were detected only in MACS and CS groups, with no significant difference observed between them (*P* > 0.05). Multiple confluent nodules were present in all *ARMC5*-mutated patients (16/16) but in fewer *ARMC5* wild-type patients (20/44), with high sensitivity and negative predictive value for the prediction of germline pathogenic mutations.

**Conclusion:**

No significant cortisol-related comorbidity differences were observed between radiologically suspected PBMAH patients with NFAT and MACS. Germline *ARMC5* screening should prioritize patients with radiological findings of multiple confluent macronodules.

**Significance statement:**

Our work provides new insights into the management of primary bilateral macronodular adrenal hyperplasia (PBMAH): i) MACS and NFAT patients with radiologically suspected PBMAH (i.e., bilateral benign adrenal macronodules) may require equal clinical attention; ii) we identified seven novel *ARMC5* pathogenic variants; iii) multiple confluent adrenal nodules on imaging demonstrate predictive value for *ARMC5* pathogenic mutations, refining genetic screening criteria.

## Introduction

Primary bilateral macronodular adrenal hyperplasia (PBMAH), also termed bilateral macronodular adrenocortical disease, is characterized by benign macronodules (≥1 cm) in both adrenal glands. PBMAH is a rare and heterogeneous disease with diverse clinical, hormonal, and imaging manifestations (1). Although histopathology remains the diagnostic gold standard, many PBMAH studies include patients with imaging evidence of bilateral adrenal macronodules and concurrent autonomous cortisol secretion due to the lack of surgical indications in most cases. Notably, PBMAH, particularly in early stages, may manifest as nonfunctional adrenal tumors (NFAT) due to slow progression and inefficient steroidogenesis (2). In addition, aldosterone co-secretion in PBMAH can present as primary aldosteronism (PA) (2). The broad phenotypic variability of PBMAH increases misdiagnosis risks, leading to underestimated prevalence.

Misdiagnosis or delayed diagnosis caused by the heterogeneity and insidiousness of PBMAH (3) subjects patients to the risk of prolonged hypercortisolism, which may trigger overt Cushing’s syndrome (CS) or cardiovascular events. Advances in imaging technology have highlighted the role of adrenal imaging features in identifying PBMAH, enabling earlier intervention. However, it remains unclear which patients with radiologically suspected PBMAH truly align with the heterogeneous spectrum of PBMAH. Germline mutations in the tumor suppressor gene *ARMC5* are a well-established etiology of PBMAH, accounting for 20–25% of sporadic cases and 80% of familial cases (4). Therefore, we analyzed clinical characteristics and *ARMC5* genotypes in patients with radiologically suspected PBMAH to confirm the diagnosis of PBMAH, at least the diagnosis of PBMAH linked to *ARMC5* mutations.

Furthermore, the term ‘mild autonomous cortisol secretion (MACS)’ has been introduced to describe patients with post-dexamethasone serum cortisol >50 nmol/L (1.8 μg/dL), who have elevated risks of cortisol-related comorbidities and mortality (5). Recent guidelines on adrenal incidentalomas emphasize that MACS, compared to NFAT, is associated with higher morbidity of hypertension, diabetes, dyslipidemia, and all-cause mortality in adrenal incidentaloma patients. The guidelines recommend screening for hypertension, diabetes, and vertebral fractures in patients with MACS and adrenal incidentaloma, alongside annual reassessment of comorbidities in non-surgical cases (5). In addition, longitudinal studies indicated that patients with MACS have higher cardiovascular event rates than those with NFAT during follow-up (6, 7, 8, 9). Bilateral adrenal nodules are less prevalent than unilateral nodules but are associated with a higher risk of autonomous cortisol secretion (7). As a clinically distinct subtype of adrenal nodules, a considerable proportion of bilateral nodules are PBMAH. We also compared comorbidities in patients with radiologically suspected PBMAH (defined as bilateral adrenal macronodules without malignancy) across different hormonal profiles to investigate whether the above recommendations for adrenal incidentalomas apply to this distinct population.

## Methods

### Patients

This cross-sectional study included all patients with ≥1 cm benign bilateral adrenal nodules identified by computed tomography and/or magnetic resonance imaging at the Chinese PLA General Hospital from March 2004 to March 2025. Exclusion criteria were as follows: i) incomplete hormonal evaluation (e.g., no dexamethasone suppression test (DST) or aldosterone-to-renin ratio (ARR) screening), use of medications affecting cortisol/aldosterone levels during assessment, or concurrent conditions such as pregnancy, liver dysfunction, or renal impairment; ii) ACTH-dependent adrenal hyperplasia (including Cushing’s disease and ectopic ACTH syndrome); non-adrenocortical lesions (e.g., malignant nodules, tuberculosis, or pheochromocytoma), congenital adrenal hyperplasia, and bilateral adenomas with imaging evidence of glandular atrophy (adrenal width <3 mm). Finally, 485 subjects with radiologically suspected PBMAH were included. The study was conducted in accordance with the Helsinki Declaration. Written informed consent was obtained for genetic analysis, and all data were de-identified.

### Clinical evaluation and definitions

**Figure 1:**
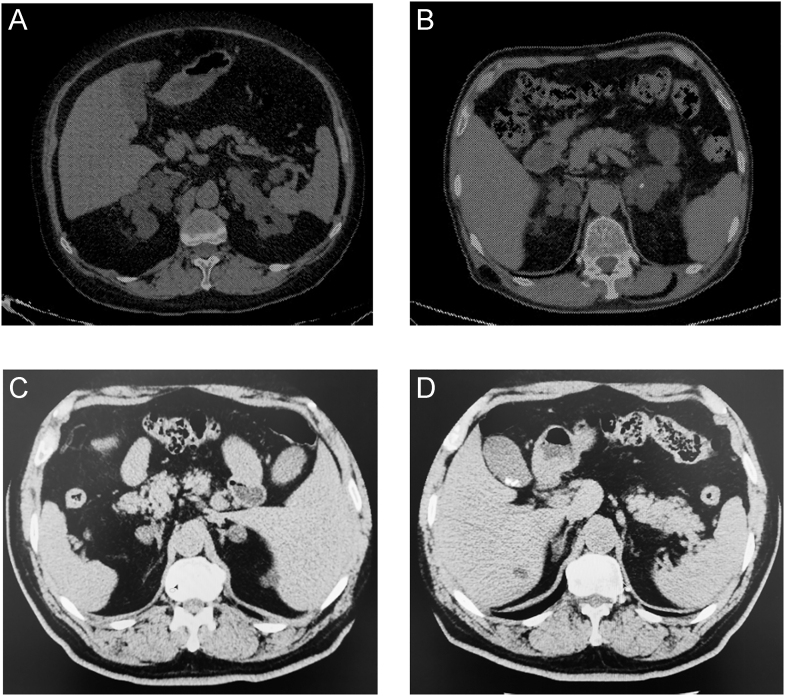
Confluent nodule: adrenal nodule coalescing with a thickened adrenal gland or adjacent nodules, typically exhibiting a ginger-like (A) or cauliflower-like (B) morphological pattern. Non-confluent nodule: well-defined and regular-shaped adrenal nodule with a normal adrenal gland. (C and D) Show bilateral multiple nodules and normal adrenal glands in an ARMC5 wild-type patient.

Clinical data were collected, including demographic characteristics, physical examinations, hormonal investigations, imaging evaluations, and comorbidity assessments at the time of diagnosis. Total adrenal mass size was defined as the sum of the diameters of the right and left major nodules. A confluent nodule was defined as a nodule coalescing with a thickened adrenal gland or adjacent nodules, typically exhibiting a ginger-like (Fig. 1A) or cauliflower-like (Fig. 1B) morphological pattern rather than well-defined, and regular-shaped nodules with normal adrenal glands (Fig. 1C and D), and was measured as one nodule (10). Imaging assessments were initially performed by an endocrinologist trained by the Department of Radiology and further reviewed by two experienced radiologists (with 13 and 12 years of specialty experience, respectively). Patients presenting with symptoms including moon face, skin fragility, sanguineous appearance, buffalo hump, truncal obesity, proximal weakness, purple striae on the skin, or hirsutism were classified as having overt Cushing’s syndrome (CS). Mild autonomous cortisol secretion was diagnosed in the presence of 1 mg-DST serum cortisol (F) > 50 nmol/L (1.8 μg/dL) without CS symptoms (5). ARR was used as a screening test to identify potential patients with PA. Patients with elevated ARR (≥3.7 ng.dL^−1^/mU.L^−1^ (30 ng.dL^−1^/(ng.mL^−1^.h^−1^))) underwent saline infusion and/or captopril challenge tests to confirm PA (11).

Hypertension was defined as systolic blood pressure (BP) ≥140 mmHg, diastolic BP ≥ 90 mmHg, or current use of antihypertensive medication. Body mass index (BMI) was calculated as weight (kg)/(height (m))^2^, with obesity defined as BMI ≥28 kg/m^2^ according to the Chinese criteria (12). Dyslipidemia was diagnosed if serum total cholesterol ≥6.2 mmol/L, low-density lipoprotein cholesterol (LDL-C) ≥4.1 mmol/L, high-density lipoprotein cholesterol (HDL-C) <1.0 mmol/L, triglycerides ≥2.3 mmol/L, or if lipid-lowering drugs were used (13, 14). Diabetes and prediabetes were diagnosed following established standard guidelines (15), and the diagnosis of diabetes was also supported by the patient’s use of glucose-lowering agents (including insulin or oral antihyperglycemic drugs). Osteoporosis was diagnosed by a bone mineral density T-score < −2.5 or a fragility fracture (16). Histories of cardio-cerebrovascular diseases were obtained from patient self-reports or the electronic medical records system.

### Germline *ARMC5* sequencing and analysis

Among the included 485 subjects with radiologically suspected PBMAH, a subgroup of 69 consecutive patients who sought medical care at our institution during the prospective recruitment period (January 2024–March 2025) was invited for genetic testing. Of these, peripheral blood leukocyte DNA samples from 62 patients who consented to genetic testing were ultimately subjected to Sanger sequencing of the coding region and flanking introns of the *ARMC5* gene, with primers and conditions described by Assié *et al.* (2). Variants were named according to the reference transcript NM_001105247.2 and verified using the HGVS nomenclature checker (Mutalyzer, https://mutalyzer.nl/name-checker). Pathogenicity of variants was evaluated based on population databases (gnomAD, https://gnomad.broadinstitute.org), variant databases (ClinVar, https://www.ncbi.nlm.nih.gov/clinvar/), computational predictions (REVEL scores (17) (https://sites.google.com/site/revelgenomics/) for missense variants (18) and SpliceAI for splicing effects (19)), family segregation, and published the literature, following the American College of Medical Genetics and Genomics/Association for Molecular Pathology (ACMG/AMP) guidelines. The application of the ACMG/AMP criteria to *ARMC5* variant classification referred to the review by Bouys *et al.* (4). Variants were categorized as benign variants, likely benign variants, variants of uncertain significance (VUS), likely pathogenic variants, or pathogenic variants. Patients without *ARMC5* variants or those harboring benign/likely benign missense or synonymous variants were classified as *ARMC5* wild-type. Patients with pathogenic or likely pathogenic variants were considered *ARMC5*-mutated type (20).

### Statistical analysis

Data were expressed as mean ± standard deviation (SD) for normally distributed continuous variables, median (interquartile range (IQR)) for non-normally distributed continuous variables, and counts (percentages) for categorical variables. For normally distributed data with homogeneity of variance, intergroup comparisons were performed using independent *t*-tests (two groups) or one-way analysis of variance (ANOVA) followed by the least significant difference (LSD) post-hoc test (multi-group). Non-normally distributed data were compared with the Mann–Whitney U test (two groups) or the Kruskal–Wallis test with Bonferroni-corrected *P*-values (multi-group). Categorical variables were compared by the Chi-square test, and intergroup differences for multiple tests were corrected using the Bonferroni method. *P* < 0.05 (two-sided) was considered statistically significant. All analyses were conducted using SPSS 26.0, except for gene visualization and receiver operating characteristic (ROC) curve analyses, which were performed with R software (version 4.3.3).

### Literature review on adrenal imaging in patients with known *ARMC5* genotype

We searched the *PubMed* database for studies on *ARMC5* published before April 1, 2025, using the search term ‘*ARMC5*’, and observed the adrenal radiological images of patients with known *ARMC5* genotype.

## Results

### Clinical characteristics of patients with radiologically suspected PBMAH

As shown in Table 1, 485 patients with radiologically suspected PBMAH were eventually included, with a mean age of 53.97 ± 9.76 years and a male-to-female ratio of 1.8:1. For endocrine functional status, MACS (41.0%) and NFAT (30.1%) were predominant, with CS accounting for 14.4%, PA for 8.9%, and coexisting PA and MACS (PA + MACS) for 5.6%. Imaging revealed that total adrenal mass size followed the order: CS group > MACS group > NFAT, PA, and PA + MACS groups. A higher proportion of multiple confluent adrenal nodules was observed in the MACS (62.3%) and CS (74.3%) groups compared to the other groups (all *P*-values Bonferroni-adjusted: MACS vs NFAT: *P* = 1.289 × 10^−14^; MACS vs PA: *P* = 1.781 × 10^−5^; MACS vs PA + MACS: *P* = 1.790 × 10^−4^; CS vs NFAT: *P* = 5.402 × 10^−14^; CS vs PA: *P* = 9.786 × 10^−7^; CS vs PA + MACS: *P* = 8.347 × 10^−5^).

**Table 1:** Clinical characteristics of patients with radiologically suspected PBMAH stratified by endocrine functional status.

	Overall	NFAT	MACS	CS	PA	PA + MACS
*n* (%)	485	146 (30.1%)	199 (41.0%)	70 (14.4%)	43 (8.9%)	27 (5.6%)
Age at diagnosis, years	53.97 ± 9.76	54.49 ± 8.68	54.67 ± 10.33	50.97 ± 11.57	53.53 ± 8.16	54.52 ± 6.98
Males, *n* (%)	314 (64.7%)	106 (72.6%)	131 (65.8%)	31 (44.3%)*^,^^†^^,^^§^	33 (76.7%)	13 (48.2%)
Investigated for, *n* (%)						
Incidentaloma	322 (66.4%)	115 (78.8%)	155 (77.9%)	25 (35.7%)*^,^^†^	16 (37.2%)*^,^^†^	11 (40.7%)*^,^^†^
HBP	93 (19.2%)	35 (17.1%)	33 (16.6%)	19 (27.1%)	9 (20.9%)	7 (25.9%)
Symptoms of CS	18 (3.7%)	0 (0%)	0 (0%)	18 (25.7%)*^,^^†^^,^^§^^,^^║^	0 (0%)	0 (0%)
Fatigue	3 (0.6%)	0 (0%)	1 (0.5%)	1 (1.4%)	0 (0%)	1 (3.7%)
Hypokalemia	45 (9.3%)	6 (4.1%)	7 (3.5%)	6 (8.6%)	18 (41.9%)*^,^^†^^,^^‡^	8 (29.6%)*^,^^†^
Family history of PBMAH	0 (0%)	0 (0%)	2 (1%)	0 (0%)	0 (0%)	0 (0%)
Osteoporosis	2 (0.4%)	0 (0%)	1 (0.5%)	1 (1.4%)	0 (0%)	0 (0%)
Total adrenal mass size, mm	53 (40.73)	44 (35.57)	62 (48.77)*^,^^§^^,^^║^	84 (54.105)*^,^^†^^,^^§^^,^^║^	37 (32.56)	43 (36.59)
Multiple confluent adrenal nodules, *n* (%)	218 (44.9%)	27 (18.5%)^†^^,^^‡^	124 (62.3%)	52 (74.3%)	9 (20.9%)^†^^,^^‡^	6 (22.2%)^†^^,^^‡^
HBP, *n* (%)	399 (82.3%)	101 (69.2%)	161 (80.9%)	67 (95.7%)*^,^^†^	43 (100%)*^,^^†^	27 (100%)*
Duration of HBP, months	84 (34.180)	84 (48.108)	84 (24.180)	60 (24.120)*^,^^║^	96 (48.156)	120 (96.192)
Antihypertensive treatment, *n* (%)	354 (88.5%)	83 (82.2%)	141 (87%)	64 (95.5%)	40 (93%)	26 (96.3%)
BMI, kg/m^2^	27.00 ± 3.43	27.18 ± 2.95	26.45 ± 3.64*	27.92 ± 3.20^†^	27.10 ± 3.64	27.55 ± 3.94
Obesity, *n* (%)	165 (34.1%)	50 (34.2%)	58 (29.1%)	30 (43.5%)	17 (39.5%)	10 (37%)
Dyslipidemia, *n* (%)	253 (52.9%)	89 (61.4%)	93 (47.9%)	36 (51.4%)	21 (50%)	14 (51.9%)
FBG, mmol/L	5.50 ± 1.58	5.46 ± 1.30	5.49 ± 1.74	5.55 ± 1.96	5.67 ± 1.30	5.39 ± 0.97
HbA1c, %	6.29 ± 1.19	6.15 ± 0.95	6.33 ± 1.28	6.64 ± 1.372^§^	6.14 ± 1.34	6.06 ± 0.82
Diabetes, *n* (%)						
No	137 (28.2%)	38 (26%)	65 (32.7%)	14 (20.0%)	14 (32.6%)	6 (22.2%)
IGT	146 (30.1%)	48 (32.9%)	55 (27.6%)	24 (34.3%)	13 (30.2%)	6 (22.2%)
Yes	202 (41.6%)	60 (41.1%)	79 (39.7%)	32 (45.7%)	16 (37.2%)	15 (55.6%)
Duration of diabetes, months	24 (3.84)	30 (6.5, 105)	24 (4.120)	18 (1.48)	36 (0.96)	21 (0.60)
Antihyperglycemic treatment, *n* (%)	159 (78.7%)	46 (76.7%)	65 (82.5%)	25 (78.1%)	13 (81.3%)	10 (66.7%)
Insulin treatment, *n* (%)	47 (23.2%)	8 (13.1%)	21 (26.6%)	10 (31.3%)	4 (25%)	4 (26.7%)
Serum potassium, mmol/L	3.76 ± 0.50	3.93 ± 0.40	3.87 ± 0.34	3.59 ± 0.58*^,^^†^	3.37 ± 0.57*^,^^†^	3.10 ± 0.58*^,^^†^^,^^‡^
History of hypokalemia, *n* (%)	156 (32.2%)	23 (15.8%)	41 (20.6%)	35 (50.0%)*^,^^†^	34 (79.1%)*^,^^†^^,^^‡^	23 (85.2%)*^,^^†^^,^^‡^
Osteoporosis, *n* (%)	68 (14%)	9 (6.2%)	29 (14.6%)	21 (30%)*^,^^†^	4 (9.3%)	5 (18.5%)
Coronary heart disease, *n* (%)	85 (17.5%)	24 (16.4%)	41 (20.6%)	9 (12.9%)	7 (16.3%)	4 (14.8%)
Cerebrovascular disease, *n* (%)						
No	427 (88%)	137 (93.8%)	172 (86.4%)	61 (87.1%)	33 (76.7%)*	24 (88.9%)
Ischemia	52 (10.7%)	9 (6.2%)	23 (11.6%)	9 (12.9%)	9 (20.9%)*	2 (7.4%)
Hemorrhage	4 (0.8%)	0 (0%)	2 (1%)	0 (0%)	1 (2.3%)	1 (3.7%)
Ischemia & hemorrhage	2 (0.4%)	0 (0%)	2 (1%)	0 (0%)	0 (0%)	0 (0%)
Germline ARMC5						
Wild type	44 (71%)	9 (100%)	21 (61.8%)	4 (44.4%)	8 (100%)	2 (100%)
Mutated type	16 (25.8%)	0 (0%)	12 (35.3%)	4 (44.4%)	0 (0%)	0 (0%)
VUS	2 (3.2%)	0 (0%)	1 (2.9%)	1 (11.1%)	0 (0%)	0 (0%)

NFAT, nonfunctioning adrenal tumor; MACS, mild autonomous cortisol secretion; CS, overt Cushing’s syndrome; PA, primary aldosteronism; PA + MACS, coexisting primary aldosteronism and mild autonomous cortisol secretion; PBMAH, primary bilateral macronodular adrenal hyperplasia; HBP, hypertension; BMI, body mass index; FBG, fasting blood glucose; HbA1c, glycated hemoglobin; IGT, impaired glucose tolerance; VUS, variants of uncertain significance.

Statistical methods for intergroup comparisons: normally distributed data, one-way analysis of variance (ANOVA) with the least significant difference (LSD) post-hoc test; non-normally distributed data, Kruskal–Wallis test with Bonferroni correction; categorical data, Chi-square test with Bonferroni adjustment. *P* < 0.05 (two-sided) was considered statistically significant. Significance markers denote pairwise comparisons.

* *P* < 0.05 vs NFAT group.

^†^ *P* < 0.05 vs MACS group.

^‡^ *P* < 0.05 vs CS group.

^§^ *P* < 0.05 vs PA group.

^║^ *P* < 0.05 vs PA + MACS group.

Compared to patients with NFAT, those with MACS had a lower BMI, with no significant differences in the prevalence and severity of comorbidities, including hypertension, obesity, dyslipidemia, diabetes, hypokalemia, osteoporosis, coronary heart disease, and cerebrovascular disease (*P* > 0.05). Hypertension prevalence was higher in the CS and PA groups than in the MACS group, and was also higher in the CS, PA, and PA + MACS groups than in the NFAT group. HbA1c was elevated in CS compared to PA, while no statistical differences were observed in blood glucose levels or diabetes-related parameters (prevalence, duration, and therapy) across functional subgroups. Serum potassium was lower in the CS, PA, and PA + MACS groups than in the NFAT and MACS groups; moreover, the PA + MACS group had lower levels than the CS group. The proportion of history of hypokalemia followed the order: PA and PA + MACS groups > CS group > NFAT and MACS groups. The prevalence of osteoporosis was higher in CS than in NFAT or MACS. No statistical differences in coronary heart disease or cerebrovascular disease were observed across functional subgroups. Analysis of cortisol-related biochemical parameters (Supplementary Table 1 (see section on Supplementary materials given at the end of the article)) showed significantly higher urinary free cortisol (UFC), midnight cortisol, and 08:00 h cortisol levels in the MACS and CS groups compared with the NFAT group (*P* < 0.05).

Among patients with radiologically suspected PBMAH, 218 patients had multiple confluent adrenal nodules (Supplementary Table 2), with MACS (56.9%) and CS (23.9%) accounting for the majority. Total adrenal mass size was larger in CS than in the other groups, and larger in MACS than in NFAT (*P* < 0.05). No statistically significant differences in other clinical characteristics (demographics, physical examination, and comorbidities) were observed between the NFAT and MACS groups in patients with multiple confluent adrenal nodules, except for a lower BMI in the MACS group, consistent with findings in the total population.

### Germline *ARMC5* variants in patients with radiologically suspected PBMAH

In 62 unrelated patients with radiologically suspected PBMAH who underwent germline *ARMC5* testing, 2 patients had no *ARMC5* mutations, 60 patients carried 30 distinct variants (Table 2), including 4 variants in the 5′ untranslated region (UTR), 1 variant in the 3′UTR, 2 intronic variants, 6 synonymous variants, and 17 amino acid-altering variants (five nonsense, seven frameshift, and five missense variants). Pathogenic mutations were identified in 16 patients, corresponding to 13 distinct pathogenic variants. Among these, the following seven pathogenic variants were not reported previously: c.2377C>T (p.Arg793*), c.1691del (p.Pro564Hisfs*66), c.2018_2019delinsC (p.Arg673Profs*16), c.2207_2216del (p.Tyr736Cysfs*178), c.220del (p.Leu74Tyrfs*63), c.256del (p.Gln86Argfs*51), and c.73del (p.Glu25Argfs*16). The 5′UTR variant c.-306T>C occurred in 54 patients (87.1%), exceeding its reported frequency of 67.5% in the East Asian population of the gnomAD database (Table 3). The intronic variant c.1864+250C>T was present in 43 patients (69.4%), significantly higher than the 46.2% frequency in the East Asian population of the gnomAD database (Table 3).

**Table 2:** Summary of all germline ARMC5 variants found in patients with radiologically suspected PBMAH.

Number of cases	Nucleotide variation	Protein variation	Variant type	Reference SNP (rs)	ACMG classification	References reported
54	c.-306T>C	-	5'UTR variant	rs3813002	Benign	/
43	c.1864+250C>T	-	Intronic variant	rs11150624	Benign	/
18	c.475+58A>G	-	Intronic variant	rs9926717	Benign	/
4	c.1084C>T	p.Arg362Trp	Missense	rs1385397608	Pathogenic	(25, 26, 27, 28, 29, 30)
3	c.969C>T	p.Gly323=	Synonymous	rs190300567	Benign	/
2	c.-247C>T	-	5'UTR variant	rs531990046	Benign	/
2	c.1317T>C	p.Phe439=	Synonymous	rs182524829	Benign	/
1	c.-400T>C	-	5'UTR variant	rs1373792876	VUS	/
1	c.-9C>G	-	5'UTR variant	rs763121282	VUS	/
1	c.*307T>C	-	3'UTR variant	rs139226061	Benign	/
1	c.1222C>T	p.Gln408*	Nonsense	rs1367418052	Pathogenic	(31) (somatic tumoral ARMC5 variants)
1	c.1288G>T	p.Glu430*	Nonsense	-	Pathogenic	(32, 33)
1	c.1855C>T	p.Arg619*	Nonsense	rs766717248	Pathogenic	(2, 31, 32, 33, 34, 35, 36)
1	c.2377C>T	p.Arg793*	Nonsense	rs1489499125	Likely pathogenic	/
1	c.799C>T	p.Arg267*	Nonsense	rs369721476	Pathogenic	(2, 32, 33, 37, 38, 39)
1	c.1691del	p.Pro564Hisfs*66	Frameshift	-	Pathogenic	/
1	c.2018_2019delinsC	p.Arg673Profs*16	Frameshift	-	Likely pathogenic	/
1	c.2207_2216del	p.Tyr736Cysfs*178	Frameshift	-	Likely pathogenic	/
1	c.220del	p.Leu74Tyrfs*63	Frameshift	-	Pathogenic	/
1	c.256del	p.Gln86Argfs*51	Frameshift	-	Pathogenic	/
1	c.294del	p.Gly99Glufs*38	Frameshift	-	Pathogenic	(36) (somatic tumoral ARMC5 variants)
1	c.73del	p.Glu25Argfs*16	Frameshift	-	Pathogenic	/
1	c.2321T>C	p.Phe774Ser	Missense	-	VUS	/
1	c.1023C>G	p.Ser341Arg	Missense	-	VUS	/
1	c.1084C>G	p.Arg362Gly	Missense	-	VUS	/
1	c.1766C>T	p.Ala589Val	Missense	rs778251907	VUS	/
1	c.1335T>C	p.Pro445=	Synonymous	rs547658734	VUS	/
1	c.1485G>A	p.Pro495=	Synonymous	rs762929772	VUS	/
1	c.1641G>A	p.Ala547=	Synonymous	rs61732352	Benign	/
1	c.1842C>G	p.Leu614=	Synonymous	rs55800131	Benign	/

**Table 3:** Allele frequency of germline ARMC5 variants in patients with radiologically suspected PBMAH and in controls from East Asian populations in the gnomAD database (v4.1.0).

ARMC5 variant	ACMG classification	ARMC5 SNP in patients (*n* = 62)	ARMC5 SNP in east Asian in the gnomAD database	P
c.-306T>C (chr16-31459219 T>C)	Benign	54 (87.1%)	27,545 (67.5%) (*n* = 40,816)	**0.001** *
c.1864+250C>T (chr16-31465137C>T)	Benign	43 (69.4%)	20,709 (46.2%) (*n* = 44,838)	**<0.001** *
c.475+58A>G (chr16-31460057 A>G)	Benign	18 (29%)	9,169 (21%) (*n* = 43,638)	0.121

* *P* < 0.05.

**Figure 2:**
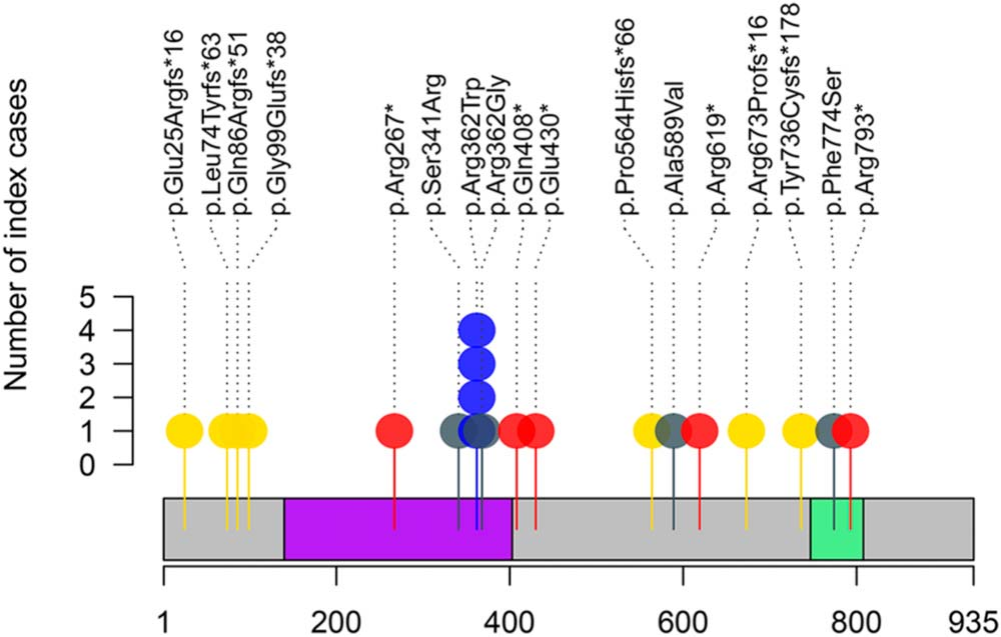
Germline ARMC5 variants in the ARMC5 protein (NM_001105247.2 canonical transcript). The Armadillo and BTB/POZ domains are depicted as purple and green regions, respectively. Class 4 (likely pathogenic) and class 5 (pathogenic) variants are color-coded as follows: nonsense (red), frameshift (gold), and missense (blue). Class 3 variants (uncertain significance) are shown in gray. The height of the lollipop tail corresponds to the number of cases in the study population.

Figure 2 illustrates variants on a graphical representation of *ARMC5* protein (reference transcript: NM_001105247.2), not including 5′ and 3′ UTR alterations, intronic variants, and synonymous variants, all of which were predicted *in silico* not to affect splicing. Pathogenic variants were distributed across the *ARMC5* coding sequence, most of which were unique to individual patients. Only p.Arg362Trp was identified in four unrelated patients. Variants p.Arg267*, p.Ser341Arg, p.Arg362Trp, and p.Arg362Gly were located in the Armadillo domain. Variants p.Phe774Ser and p.Arg793* were situated in the BTB/POZ domain.

### Phenotypes and *ARMC5* genotypes

Phenotype analysis included 16 *ARMC5*-mutated patients and 44 *ARMC5* wild-type patients, with two additional patients excluded due to uncertain *ARMC5* genotypes from carrying VUS (Table 1). Pathogenic mutations were absent in the NFAT, PA, and PA + MACS groups but were detected in the MACS and CS groups. However, no significant difference in mutation frequency was observed between the MACS (12/35, 34.3%) and CS (4/8, 50%) subgroups (*P* > 0.05) (Table 4).

**Table 4:** Germline ARMC5 mutations in radiologically suspected PBMAH: cortisol secretion status stratification.

	Overall	Absence of cortisol secretion	MACS	CS
ARMC5 wild-type	44 (73.3%)	17 (100%)	23 (65.7%)*	4 (50%)*
ARMC5-mutated type	16 (26.7%)	0 (0%)	12 (34.3%)*	4 (50%)*

* *P* < 0.05 versus absence of cortisol secretion.

Compared to *ARMC5* wild-type patients, *ARMC5*-mutated patients exhibited higher midnight serum cortisol levels, more suppressed ACTH (<2.2 pmol/L), and larger adrenal mass size (*P* < 0.05). Cortisol-related comorbidity prevalence did not differ statistically between the two groups (*P* > 0.05). Notably, all *ARMC5*-mutated patients (16/16, 100%) displayed autonomous cortisol secretion and imaging evidence of multiple confluent adrenal nodules (Table 5 and Supplementary Table 3). In contrast, these features were less common in *ARMC5* wild-type patients (autonomous cortisol secretion: 25/44, 56.8%; multiple confluent nodules: 20/44, 45.5%). Sensitivity, specificity, and positive and negative predictive values for the prediction of germline ARMC5 pathogenic mutations are shown in Table 6. ACTH_08:00_ < 2.2 pmol/L, total adrenal nodule size, and the presence of multiple confluent adrenal nodules were predictive markers achieving an area under the curve (AUC) > 0.7. For screening purposes, high-sensitivity markers are prioritized. Both unsuppressed F after low-dose dexamethasone suppression testing (LDDST) and multiple confluent adrenal nodules achieved high sensitivity and negative predictive value in this preliminary analysis.

**Table 5:** Comparisons between ARMC5 wild-type and ARMC5-mutated patients with radiologically suspected PBMAH.

	ARMC5 wild-type	ARMC5-mutated type	P
*n* (%)	44 (73.33%)	16 (22.86%)	
Age at diagnosis, years	55.55 ± 7.86	54.25 ± 9.64	0.598
Males, *n* (%)	31 (70.5%)	11 (68.8%)	1
Investigated for, *n* (%)			0.221
Incidentaloma	30 (68.2%)	13 (81.3%)	
Hypertension	7 (15.9%)	1 (6.3%)	
Symptoms of CS	2 (4.5%)	1 (6.3%)	
Fatigue	0 (0%)	1 (6.3%)	
Hypokalemia	5 (11.4%)	0 (0%)	
24-hUFC, fold ULN	0.97 (0.70, 1.38)	1.46 (0.81, 2.31)	0.108
F_00:00_, nmol/L	116.2 (75.395, 185.43)	198.19 (126.41, 290.43)	0.045*
ACTH_08:00_ < 2.2 pmol/L	15 (34.1%)	13 (81.3%)	0.001*
F_08:00_, nmol/L	372.15 (306.48, 448.2)	376.1 (319.05, 471.54)	0.867
Endocrine functional status			0.019*
NFAT	9 (20.5%)	0 (0%)	
MACS	21 (47.7%)	12 (75%)	
CS	4 (9.1%)	4 (25%)	
PA	8 (18.2%)	0 (0%)	
PA + MACS	2 (4.5%)	0 (0%)	
Total adrenal mass size, mm	53.8 (45.72, 69.70)	75.06 (68.97, 82.97)	0.003*
Multiple confluent adrenal nodules, *n* (%)	20 (45.5%)	16 (100%)	<0.001*
Hypertension, *n* (%)	38 (86.4%)	14 (87.5%)	1
BMI, kg/m^2^	26.34 ± 3.14	26.20 ± 3.35	0.876
Obesity, *n* (%)	12 (27.3%)	4 (25%)	1
Dyslipidemia, *n* (%)	23 (53.5%)	6 (37.5%)	0.275
FBG, mmol/L	5.66 ± 1.23	5.76 ± 3.59	0.921
HbA1c, %	6.65 ± 1.22	7.23 ± 2.86	0.503
Diabetes, *n* (%)			0.089
No	9 (20.5%)	7 (43.8%)	
IGT	8 (18.2%)	4 (25%)	
Yes	27 (61.4%)	5 (31.3%)	
Serum potassium, mmol/L	3.812 ± 0.538	3.77 ± 0.374	0.741
History of hypokalemia, *n* (%)	18 (40.9%)	5 (31.3%)	0.145
Osteoporosis, *n* (%)	7 (15.9%)	3 (18.8%)	1
Coronary heart disease, *n* (%)	10 (22.7%)	1 (6.3%)	0.259
Cerebrovascular disease, *n* (%)			1
No	38 (86.4%)	15 (93.8%)	
Ischemia	4 (9.1%)	1 (6.3%)	
Hemorrhage	2 (4.5%)	0 (0%)	

CS, Cushing’s syndrome; 24-h UFC, 24-h urinary free cortisol; ULN, upper limit of normal; F, serum cortisol; ACTH, adrenocorticotropic hormone; NFAT, nonfunctioning adrenal tumor; MACS, mild autonomous cortisol secretion; PA, primary aldosteronism; PA + MACS, coexisting primary aldosteronism and mild autonomous cortisol secretion; BMI, body mass index; FBG, fasting blood glucose; HbA1c, glycated hemoglobin; IGT, impaired glucose tolerance.

* *P* < 0.05.

**Table 6:** Sensitivity, specificity, positive and negative predictive values for the prediction of germline ARMC5 pathogenic mutations.

	AUC (95%CI)	Cutoff	Sensitivity	Specificity	PPV	NPV
F_00:00,_ nmol/L	0.67 (0.50–0.82)	164.15 nmol/L	62.5% (35.43–84.80%)	72.73% (57.21–85.04%)	45.45% (24.39–67.79%)	84.21% (68.75–93.98%)
ACTH_08:00_ < 2.2 pmol/L	0.71 (0.57–0.84)	/	75% (47.62–92.73%)	65.91% (50.08–79.51%)	44.44% (25.48–64.67%)	87.88% (71.80–96.60%)
Unsuppressed F after LDDST	0.69 (0.62–0.77)	/	100% (79.41–100%)	38.64% (24.36–54.50%)	37.21% (22.98–53.27%)	100% (80.49–100%)
Total adrenal nodule size, mm	0.78 (0.62–0.93)	64.23 mm	84.62% (54.55–98.08)	74.42% (58.83–86.48%)	50% (28.22–71.78%)	94.12% (80.32–99.28%)
Multiple confluent adrenal nodules	0.77 (0.70–0.85)	/	100% (79.41–100%)	54.55% (38.85–69.61%)	44.44% (27.93–61.90%)	100% (85.75–100%)

Data are presented as % (95% CI).

F, serum cortisol; ACTH, adrenocorticotropic hormone; LDDST, low-dose dexamethasone suppression test; NPV, negative predictive value; PPV, positive predictive value.

### Literature review on adrenal imaging in patients with known *ARMC5* genotype

Twenty-nine studies displayed adrenal radiological images of patients with known *ARMC5* genotype (Supplementary Table 4): all index *ARMC5*-mutated patients exhibited multiple confluent adrenal nodules. Some *ARMC5*-mutated carriers who were family members of probands presented with normal adrenal morphology, an isolated nodule, or unilateral hyperplasia, with or without autonomous cortisol secretion. *ARMC5* wild-type patients exhibited heterogeneous imaging patterns, such as a single nodule on each adrenal, an isolated nodule, or multiple confluent adrenal nodules.

## Discussion

PBMAH, a rare and heterogeneous adrenal disease, remains diagnostically challenging. Its molecular origins have only been partially elucidated since the first description of *ARMC5* pathogenic variants in 2013 (21). Many studies on PBMAH have focused on patients with bilateral adrenal macronodules accompanied by autonomous cortisol secretion, possibly because the clinical diagnosis of PBMAH is reliable in cases with autonomous cortisol secretion according to the histopathological experience, or because CS symptoms or cortisol-related comorbidities are the primary clinical concern, or because *ARMC5*-mutated cases typically present with autonomous cortisol secretion. In this study, *ARMC5* mutations were exclusively identified in patients with autonomous cortisol secretion, while absent in patients with NFAT or PA. However, current studies indicate that *ARMC5* mutations account for only approximately 20% of PBMAH index cases, with *KDM1A* mutations accounting for less than 5% (4). Consequently, over 75% of cases currently lack identifiable molecular causes, necessitating histopathologic confirmation for definitive diagnosis. Importantly, regardless of whether patients with bilateral macronodules are diagnosed with non-functional tumors, PBMAH, or PA, clinical concerns focus on the occurrence and severity of comorbidities such as hypertension, diabetes, and cardiovascular diseases. Recent guidelines on adrenal incidentalomas recommend active screening and follow-up for comorbidities in patients with MACS and adrenal incidentalomas (5). This raises the question of whether these recommendations apply to patients with radiologically suspected PBMAH. In our analysis of such patients, MACS and NFAT patients showed no significant differences in metabolic comorbidities, contrasting with previous studies primarily focused on unilateral adrenal incidentalomas (5). This discrepancy may be attributed to the unique nature of bilateral macronodular disease – including its slow progression and distinct molecular mechanisms (e.g., specific gene mutations, aberrant expression of G-protein-coupled receptors, and paracrine intra-adrenal ACTH secretion) (22). Notably, MACS patients with bilateral large nodules demonstrated significantly higher UFC, midnight cortisol, and 08:00 h cortisol levels than NFAT patients in our study. The lack of correlation between cortisol-related biomarkers and metabolic comorbidities implies that metabolic comorbidities in this population may stem from long-term cortisol exposure rather than short-term cortisol levels, which is consistent with PBMAH’s slow-progression nature. This supports the hypothesis that chronic low-level cortisol hypersecretion during the gradual growth of bilateral nodules induces cumulative metabolic abnormalities, with metabolic comorbidities preceding overt MACS. The comparable comorbidity profiles between MACS and NFAT groups suggest that both patient groups with radiologically suspected PBMAH require equivalent clinical vigilance, distinct from typical unilateral adrenal incidentalomas. In our study of patients with radiologically suspected PBMAH, MACS subjects exhibited lower BMI than NFAT subjects, consistent with recent studies on adrenal incidentalomas (23, 24). Possible explanations include prolonged serum half-life of dexamethasone in obese patients resulting in greater HPA axis suppression, or heightened central glucocorticoid sensitivity at hypothalamic-pituitary levels in this population (23). In addition, metabolic comorbidities and BMI are influenced by multiple factors, such as genetic predisposition and lifestyle factors, not exclusively attributable to abnormal cortisol secretion. Future multicenter cohorts or prospective studies are required to further evaluate the clinical implications of MACS and long-term cardiovascular/metabolic risks in bilateral benign adrenal macronodules or PBMAH.

As mentioned earlier, the role of *ARMC5* mutations in PBMAH has been well established. PBMAH patients are considered to carry germline heterozygous inactivating variants, and their adrenal tissues suffer from a second somatic hit, which results in bi-allelic inactivation of *ARMC5* and the development of PBMAH (2, 4). To date, more than 140 distinct *ARMC5* germline pathogenic variants have been described (4), distributed across the entire *ARMC5* coding sequence, with most being private variants and only a few identified hotspots (3). The variant c.1084C>T(p.Arg362Trp) was identified in four unrelated patients in our study and was previously reported in four additional PBMAH patients (25, 26, 27, 28, 29, 30), suggesting a mutational hotspot in PBMAH. Similarly, the following variants identified in our patients are potential hotspots: c.1855C>T(p.Arg619*) (2, 31, 32, 33, 34, 35, 36) and c.799C>T(p.Arg267*) (2, 32, 33, 37, 38, 39), reported in nine patients; c.1288G>T(p.Glu430*), reported in two patients (32, 33). The variants c.1222C>T(p.Gln408*) (31) and c.294del(p.Gly99Glufs*38) (36) were previously detected only in adrenal tissues, but we identified germline variants in our patients.

In addition, our study identified seven novel pathogenic variants, including a nonsense variant c.2377C>T(p.Arg793*) and frameshift variants: c.1691del (p.Pro564Hisfs*66), c.2018_2019delinsC(p.Arg673Profs*16), c.2207_2216del(p.Tyr736Cysfs*178), c.220del(p.Leu74Tyrfs*63), c.256del(p.Gln86Argfs*51), and c.73del(p.Glu25Argfs*16).

We also found four missense variants not reported before. Given that missense variants alter a single amino acid and affect protein structure less severely than frameshift or nonsense variants in most cases, their pathogenic classification could be challenging. These four novel missense variants were classified as VUS according to the ACMG/AMP guidelines due to insufficient evidence from familial segregation, *in vitro* studies, or reported cases. However, the missense variants located in specific structural domains have drawn our attention.

*ARMC5* contains the Armadillo domain at the amino-terminal end and BTB/POZ domain at the carboxy-terminal end, both of which are highly conserved and can serve as docking platforms for many proteins (40). The ARM repeat-containing proteins (ARMCs) are widely distributed in eukaryotes and mediate protein–protein interactions (41). The canonical ARMC protein β-catenin, an important adhesion protein and signaling protein, consists primarily of an armadillo repeat domain, which binds many interaction partners. The Armadillo domain of *ARMC5* shares structural similarities with β-catenin, suggesting functional similarity (3). Therefore, this domain may be a key interaction hub to achieve specific functions for *ARMC5*. The BTB/POZ domain interacts with cullin 3 to regulate the turnover of *ARMC5* and other proteins by ubiquitination and further degradation via the proteasome pathway (4, 40). Cavalcante *et al.* (42) demonstrated that the pathogenic missense variant in the BTB/POZ domain disrupted this interaction. Therefore, experimental data are warranted to assess the role of missense variants c.1023C>G(p.Ser341Arg) and c.1084C>G(p.Arg362Gly) in the Armadillo domain, and c.2321T>C(p.Phe774Ser) in the BTB/POZ domain, which may allow a better classification for pathogenicity.

Notably, 87.1% of our detected patients carried the 5′UTR variant c.−306T>C and 69.4% carried the intronic variant c.1864+250C>T. These two variants were classified as benign according to the ACMG/AMP guidelines due to their frequencies in the general population database exceeding 5% (meeting the BA1 criterion), their presence in asymptomatic adults (BS2 criterion, although adrenal imaging confirmation was unavailable), and lack of impact on protein structure (BP7 criterion). Nevertheless, their frequencies in patients with radiologically suspected PBMAH were significantly higher than those in the East Asian population from the population database, suggesting a potential association with this specific morphological phenotype. Although sequences in the 5′UTR and intron regions do not encode proteins, these regions regulate gene expression. This finding may inspire future investigations into the effects of variants in non-coding regions on adrenal glands.

It has been noted that *ARMC5* mutations are associated with bilateral adrenal involvement, larger adrenal glands, more suppressed ACTH, and higher cortisol levels (27, 29, 32, 33), which is consistent with our observations. While current studies recommend germline *ARMC5* testing for index patients with bilateral adrenal macronodules and autonomous cortisol secretion (29, 33, 43), our study highlights the role of lesion morphology in narrowing the screening scope. PBMAH exhibits a heterogeneous morphological spectrum, varying from thickened adrenal glands with numerous nodules, a unique nodule on each adrenal gland, to diffuse hyperplasia without clearly visible nodules (1). The typical adrenal imaging exhibits ginger-like (Fig. 1A) or cauliflower-like (Fig. 1B) changes, characterized by multiple confluent nodules.

In our study, multiple confluent adrenal nodules were present in all *ARMC5*-mutated patients but in fewer ARMC5 wild-type patients. To validate this association between multiple confluent adrenal nodules and *ARMC5* mutations, we reviewed adrenal radiological imaging of individuals with known *ARMC5* genotypes from all published studies retrieved from PubMed (Supplementary Table 4), confirming that all reported *ARMC5*-mutated index cases (sporadic cases and familial probands) exhibited this phenotype based on the adrenal imaging provided. However, some *ARMC5*-mutated familial carriers exhibited heterogeneous adrenal imaging (normal morphology, isolated nodules, or unilateral hyperplasia) with or without autonomous cortisol secretion, which may be due to the absence of a somatic second-hit mutation or early-stage lesions. Our radiological observations align with the pathological classification by Violoncello *et al.* (44), which divided PBMAH into four subtypes based on pathological architecture and cell type proportions: subtypes 1, 2, and 3 exhibit coalescent adrenal nodules macroscopically (with *ARMC5* mutations frequent in subtype 1 (14/17), and rare in subtype 3 (1/8)), while subtype 4 exhibits discrete nodules separated by non-nodular adrenal regions (no *ARMC5* mutations detected (0/5)). Therefore, we hypothesize that adrenal cells with *ARMC5* pathogenic mutations grow and proliferate in a distinct pattern. Although no criterion can accurately predict *ARMC5* pathogenic mutations due to phenotypic heterogeneity (33), our findings have at least demonstrated a higher proportion of germline *ARMC5* pathogenic mutations in patients with multiple confluent adrenal nodules on imaging than in others. ROC analysis revealed that multiple confluent adrenal nodules exhibit lower specificity but higher sensitivity in predicting germline *ARMC5* mutations compared to total adrenal nodule size, suppressed ACTH (ACTH <2.2 pmol/L), and midnight serum cortisol. The high sensitivity and negative predictive value with no missed *ARMC5*-mutated cases in our preliminary analysis supported prioritizing germline *ARMC5* genetic screening in patients with multiple confluent macronodules on adrenal imaging. While unsuppressed cortisol after LDDST also showed extremely high sensitivity, its specificity for predicting germline ARMC5 mutations was lower than that of multiple confluent adrenal nodules. Further large-scale studies are needed to determine whether genetic testing should be limited to index patients with both multiple confluent adrenal macronodules and autonomous cortisol secretion, alongside all familial cases.

The limitations of our study include its single-center design and the lack of assessment of illegitimate receptor expression or other potential genetic alterations. Due to limited tissue availability and clinical feasibility, the inability to assess somatic *ARMC5* alterations (e.g., LOH or secondary mutations) in adrenal tissue restricted validation of the pathogenic mechanism of *ARMC5* in our study. In addition, only patients who recently consented to genetic testing received germline *ARMC5* testing, which may lead to potential selection bias, including temporal bias and consent-related participation bias. Although the high informed consent rate (90%) mitigated the risk of selection bias to some extent, the lack of temporal representativeness could lead to results reflecting the characteristics of recent patients. Furthermore, multiple confluent nodules are a qualitative indicator, which is not as objective and easily interpretable as other quantitative indicators including adrenal nodule size and hormonal parameters. Although imaging assessments were trained and reviewed by experienced radiologists, blinding was not implemented, and inter-rater agreement was not evaluated. In addition, multiple confluent nodules may be larger in size compared to other nodular subtypes, but we could not evaluate the effect of nodule size on the association between *ARMC5* mutations and multiple confluent nodules due to limitations in sample size.

In conclusion, this study found no significant differences in comorbidities between radiologically suspected PBMAH patients with NFAT and MACS. Germline *ARMC5* screening should prioritize index patients with imaging findings of multiple adrenal confluent macronodules.

## Supplementary materials



## Declaration of interest

The authors declare that there is no conflict of interest that could be perceived as prejudicing the impartiality of the work reported.

## Funding

This work was supported by the National Key Research and Development Plan of China, Major Project of Prevention and Treatment for Common Diseases (2022YFC2505300, sub-project: 2022YFC2505301).

## Data availability

The datasets used and analyzed during the current study are available from the corresponding author on reasonable request.

## Ethics approval

The study was conducted in accordance with the Helsinki Declaration and approved by the Ethics Committee of Chinese PLA General Hospital (No. S2023-320-02). Written informed consent was obtained for genetic analysis, and all data were de-identified.
