# Supplementary material for: Clinical heterogeneity and imaging-driven genetic screening priorities in patients with radiologically suspected primary bilateral macronodular adrenal hyperplasia
Source: Endocr Connect. 2025 Oct 22;14(10):e250290. doi: 10.1530/EC-25-0290 (PMC12552835; doi:10.1530/EC-25-0290)
Supplement: Supplementary file 1 [file supplementary_materials.pdf]

Supplementary Materials

**Supplementary Table 1. Cortisol-related biochemical parameters in patients with radiologically suspected PBMAH stratified by endocrine functional status.**	**Page 2**
**Supplementary Table 2. Clinical characteristics of patients with multiple confluent adrenal nodules.**	**Page 3-4**
**Supplementary Table 3. Clinical data of the germline ARMC5-mutated patients.**	**Page 5-15**
**Supplementary Table 4. Literature review on adrenal imaging in patients with known ARMC5 genotype**	**Page 16-31**

Supplementary Table 1. Cortisol-related biochemical parameters in patients with radiologically suspected PBMAH stratified by endocrine functional status.

	NFAT	MACS	CS	PA	PA+MACS
n (%)	146(30.1%)	199(41.0%)	70(14.4%)	43(8.9%)	27(5.6%)
UFC, nmol/L	356.5(253.8,510.5)	445.5(321.4,609.1) [n]	1417.95(729.05,1937) [n,m,p,pm]	417(249.95,537.6)	478.2(331.68,682/5)
$F_{0:00}$, nmol/L	70.97(48.45,108.39)	129.41(91.34,193.22)[n,p]	468.40(325.49,598.76)[n,m,p]	81.7(47.15,112.86)	107.41(70.41,201.27)[p]
$F_{8:00}$, nmol/L	357(273.17,435.51)	400.78(320.42,496.22)[n]	568.41(470.40,684.11)[n,m,p,pm]	349.1(265.9,451.6)	428.91(340.9,499.68)

NFAT, nonfunctioning adrenal tumor; MACS, mild autonomous cortisol secretion; CS, overt Cushing's syndrome; PA, primary aldosteronism; PA+MACS, coexisting primary aldosteronism and mild autonomous cortisol secretion; PBMAH, primary bilateral macronodular adrenal hyperplasia.

Intergroup comparisons were analyzed using the Kruskal-Wallis test with Bonferroni correction. Statistical significance was defined as $P < 0.05$ (two-sided). Significance markers denote pairwise comparisons: [n]: $P < 0.05$ vs NFAT group; [m]: $P < 0.05$ vs MACS group; [p]: $P < 0.05$ vs PA group; [pm]: $P < 0.05$ vs PA+MACS group.

Supplementary Table 2. Clinical characteristics of patients with multiple confluent adrenal nodules.

	Overall	NFAT	MACS	CS	PA	PA+MACS
n (%)	218	27(12.4%)	124(56.9%)	52(23.9%)	9(4.1%)	6(2.8%)
Age at diagnosis, years	54.21±10.07	54.74±10.34	54.81±10.17	52.35±10.75	55.67±5.90	53.33±3.20
Males, n (%)	144(66.1%)	22(81.5%)	84(67.7%)	25(48.1%) [n]	9(100%)	4(66.7%)
Investigated for, n (%)						
Incidentaloma	144(66.1%)	21(77.8%)	99(79.8%)	17(32.7%) [n,m]	5(55.6%)	2(33.3%)
HBP	42(19.3%)	6(22.2%)	20(16.1%)	13(25%)	1(11.1%)	2(33.3%)
Symptoms of CS	14(6.4%)	0(0%)	0(0%)	14(26.9%) [n,m]	0(0%)	0(0%)
Fatigue	2(0.9%)	0(0%)	1(0.8%)	1(1.9%)	0(0%)	0(0%)
Hypokalemia	13(6%)	0(0%)	2(1.6%)	6(11.5%) [m]	3(33.3%) [n,m]	2(33.3%) [n,m]
Family history of PBMAH	2(0.9%)	0(0%)	2(1.6%)	0(0%)	0(0%)	0(0%)
Osteoporosis	1(0.5%)	0(0%)	0(0%)	1(1.9%)	0(0%)	0(0%)
Total adrenal nodule size, mm	73(59,89)	59(48,70)	72(60,84) [n]	100(82,117) [n,m,p,pm]	61(55,70)	67(59,75)
HBP, n (%)	185(84.9%)	18(66.7%)	103(83.1%)	49(94.2%) N	9(100%)	6(100%)
Duration of HBP,months	72(24,162)	54.5(23,144)	72(24,180)	60(24,120)	120(84,240)	120(88.5,204)
Antihypertensive treatment, n (%)	165(75.7%)	14(51.9%)	91(73.4%)	46(88.5%) [n]	8(88.9%)	6(100%)
BMI, kg/m^2	27.14±3.41	27.88±2.63	26.35±3.41 [n]	27.87±3.14 [m,pm]	28.33±3.52	32.04±2.66 [n,m,c,p]
Obesity, n (%)	76(35%)	9(33.3%)	33(26.6%)	22(43.1%)	6(66.7%)	6(100%) N,M
Dyslipidemia, n (%)	104(49.1%)	15(55.6%)	55(46.2%)	24(46.2%)	5(62.5%)	5(83.3%)
FBG, mmol /L	5.45±1.80	5.35±1.30	5.50±1.94	5.39±1.86	5.76±1.31	5.11±0.85
HbA1c, %	6.39±1.34	6.20±1.15	6.36±1.40	6.62±1.37	6.43±1.20	5.76±0.46
Diabetes, n (%)						
No	68(31.2%)	7(25.9%)	44(35.5%)	12(23.1%)	4(44.4%)	1(16.7%)
IGT	60(27.5%)	9(33.3%)	33(26.6%)	16(30.8%)	1(11.1%)	1(16.7%)

Yes	90(41.3%)	11(40.7%)	47(37.9%)	24(46.2%)	4(44.4%)	4(66.7%)
Duration of diabetes,months	24(4,65)	60(12,120)	33(4,120)	21(1,45)	1(0,46)	12(1,50)
Antihyperglycemic treatment, n (%)	76(84.4%)	10(90.9%)	42(89.4%)	19(79.2%)	3(75%)	2(50%)
Insulin treatment, n(%)	26(28.9%)	2(18.2%)	14(29.8%)	8(33.3%)	1(25%)	1(25%)
Serum potassium,mmol/L	3.77±0.45	3.95±0.41	3.86±0.32	3.52±0.59 [n,m]	3.68±0.37	3.22±0.31 [n,m]
History of hypokalemia, n(%)	66(30.3%)	4(14.8%)	22(17.7%)	28(53.8%) [n,m]	6(66.7%) [n,m]	6(100%)) [n,m]
Osteoporosis,n(%)	35(16.1%)	2(7.4%)	16(12.9%)	16(30.8%)	1(11.1%)	0(0%)
Coronary heart disease, n(%)	40(18.3%)	5(18.5%)	26(21%)	6(11.5%)	2(22.2%)	1(16.7%)
Cerebrovascular disease, n(%)						
No	190(87.2%)	24(88.9%)	108(87.1%)	46(88.5%)	6(66.7%)	6(100%)
Ischaemia	24(11%)	3(11.1%)	13(10.5%)	6(11.5%)	2(22.2%)	0(0%)
Haemorrhage	3(1.4%)	0(0%)	2(1.6%)	0(0%)	1(11.1%)	0(0%)
Ischaemia & haemorrhage	1(0.5%)	0(0%)	1(0.8%)	0(0%)	0(0%)	0(0%)
Germline ARMC5						
Wild type	20(55.6%)	3(100%)	10(45.5%)	2(33.3%)	5(100%)	
Mutated type	16(44.4%)	0(0%)	12(54.5%)	4(66.7%)	0(0%)	

NFAT, nonfunctioning adrenal tumor; MACS, mild autonomous cortisol secretion; CS, overt Cushing's syndrome; PA, primary aldosteronism; PA+MACS, coexisting primary aldosteronism and mild autonomous cortisol secretion; PBMAH, primary bilateral macronodular adrenal hyperplasia; HBP, hypertension; BMI, body mass index; FBG, fasting blood glucose; HbA1c, glycated hemoglobin; IGT, impaired glucose tolerance.

Statistical methods for intergroup comparisons: Normally distributed data: one-way analysis of variance (ANOVA) with the least significant difference (LSD) post-hoc test; Non-normally distributed data: Kruskal-Wallis test with Bonferroni correction; Categorical data: Chi-square test with Bonferroni adjustment. P <0.05 (two-sided) was considered statistically significant. Significance markers denote pairwise comparisons: [n]: *P* <0.05 vs NFAT group; [m]: P <0.05 vs MACS group; [c]: P <0.05 vs CS group; [p]: P <0.05 vs PA group; [pm]: P <0.05 vs PA+MACS group.

Supplementary Table 3. Clinical data of the germline ARMC5-mutated patients.

Abbreviations: M, male; F, female; CS, overt Cushing syndrome; MACS, mild autonomous cortisol secretion; Y, yes: N, no; NA, not available; IGT, impaired glucose tolerance.

ID	Age at diagnosis, years	Sex	BMI,kg/m^2	Pathogenic variants	Adrenal imaging	Total adrenal nodule size, mm	Endocrine functional status	Hypertension	Diabetes	Dyslipidemia	History of hypokalemia	Osteoporosis	Coronary heart disease	Cerebrovascular disease
P1	58	M	29.75	c.1084C>T p.Arg362Trp	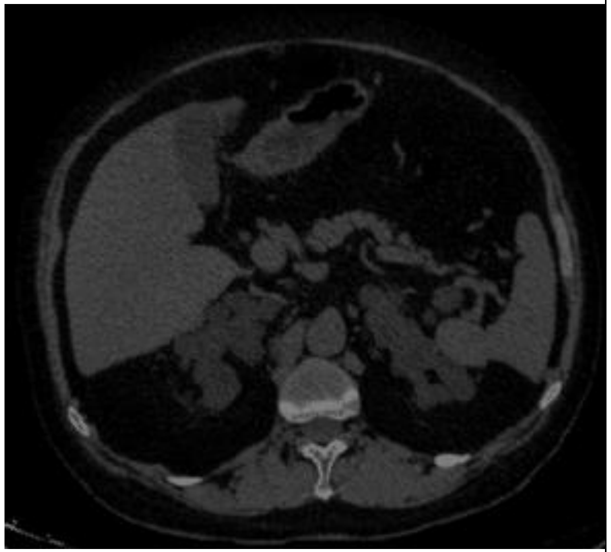	159	CS	Y	IGT	Y	Y	Y	N	N

P2	45	F	21.94	c.1855C>T<br>p.Arg619*	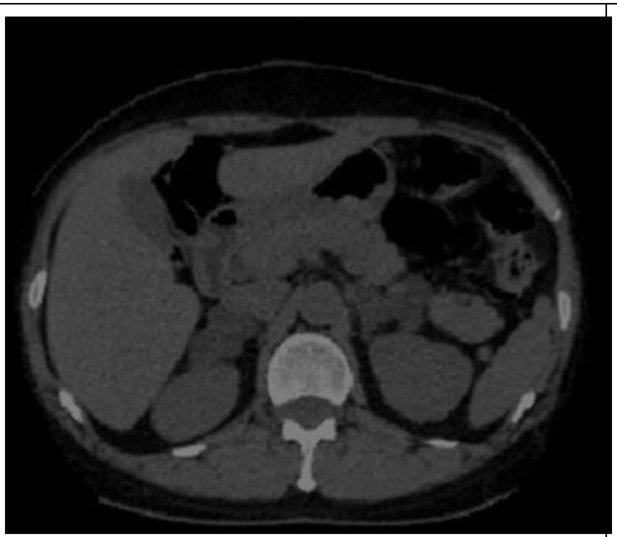	77	MACS	N	N	N	N	N	N	N
P3	66	M	27.17	c.2018_2019delinsC<br>p.Arg673Profs*16	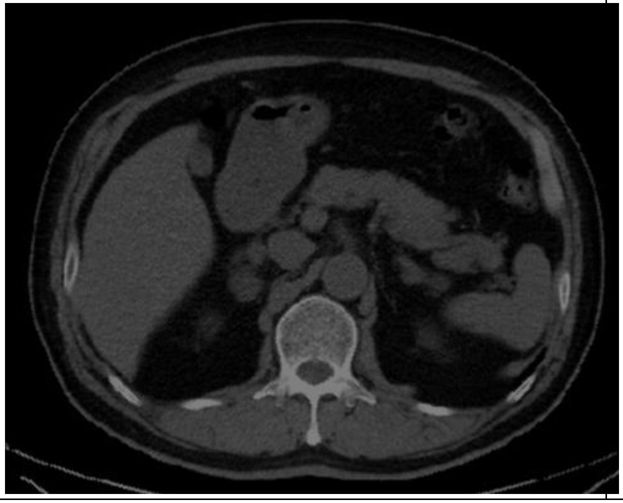	75	MACS	Y	IGT	N	N	N	N	N

P4	47	M	26.03	c.799C>T p.Arg267*	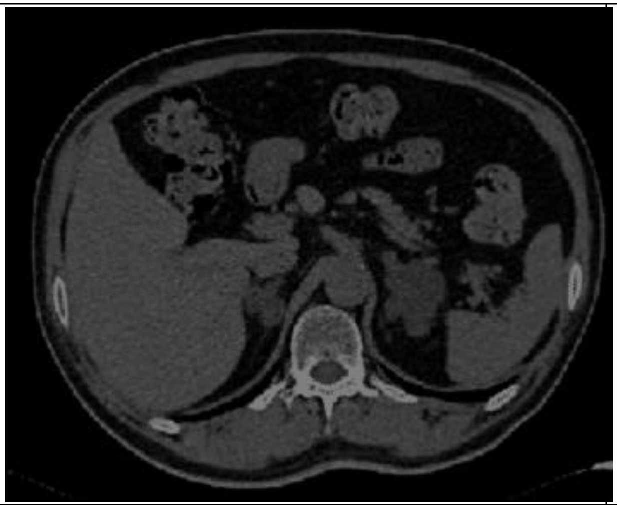	73	CS	Y	Y	Y	N	N	N	N
P5	67	F	25.88	c.1084C>T p.Arg362Trp	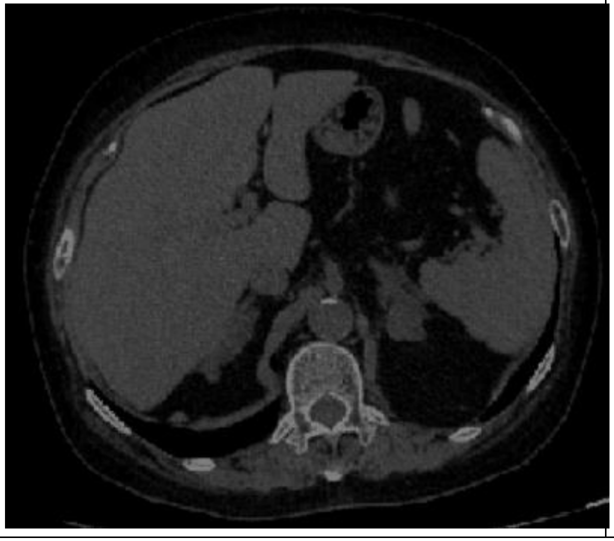	65	MACS	Y	IGT	Y	Y	Y	N	N

P6	41	M	34.40	c.1288G>T<br>p.Glu430*	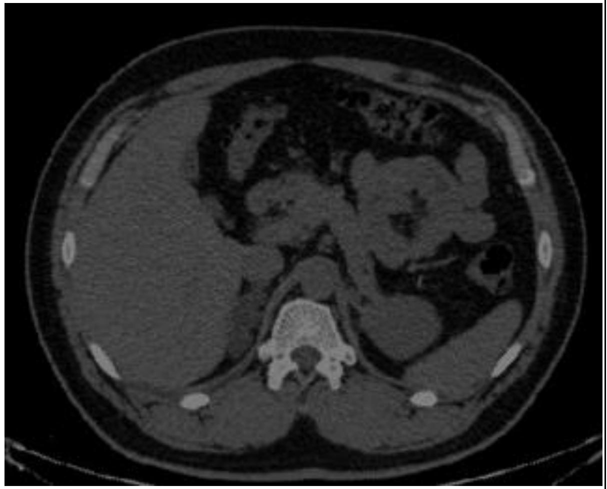	NA	MACS	Y	N	N	N	N	N	N
P7	56	F	27.39	c.1691del<br>p.Pro564Hisfs*66	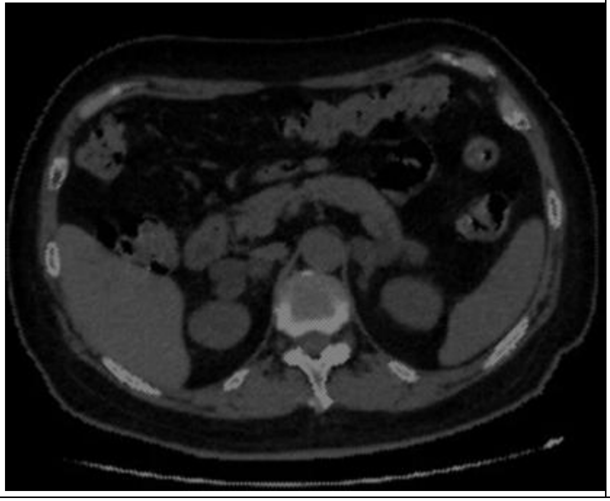	81	MACS	Y	IGT	N	N	Y	N	N

P8	37	M	30.39	c.2207_2216del p.Tyr736Cysfs*178	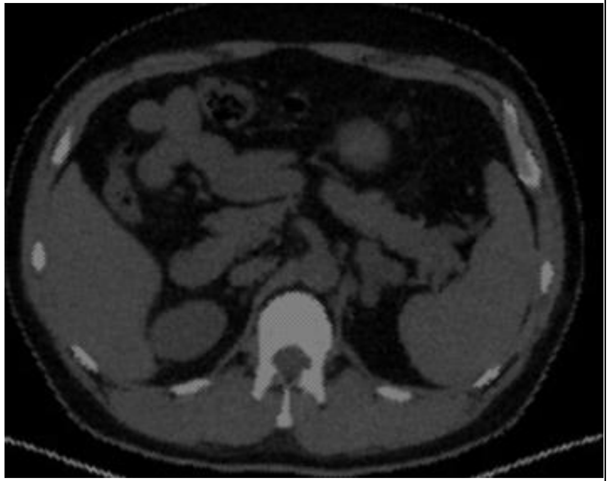 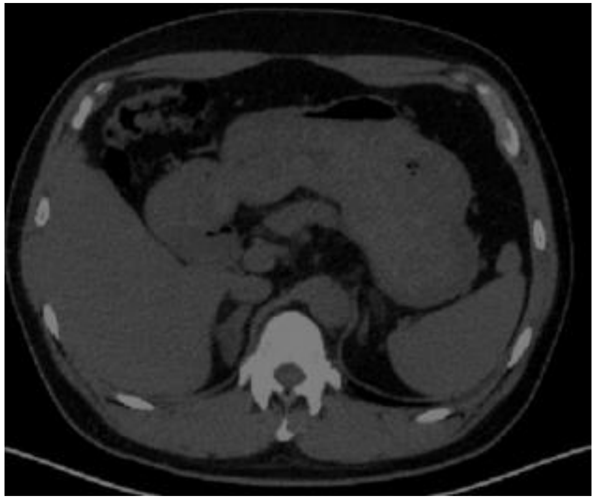	79	CS	Y	N	Y	Y	N	N	N
P9	57	M	24.73	c.220del p.Leu74Tyrfs*63	The CT report describes abnormal bilateral adrenal morphology with ginger-like architectural distortion.	26	MACS	Y	Y	N	N	N	N	N

					Multiple hypodense nodules (≤13 mm) demonstrate homogeneous precontrast CT attenuation (-7 HU). Postcontrast enhancement shows marked homogeneous arterial-phase enhancement (20 HU) and persistent portal venous phase enhancement (33 HU). Imaging documentation remains unavailable in the electronic imaging archives, as the examination exceeds 10-year archival retention thresholds.									
P10	48	M	24.54	c.1222C>T p.Gln408*	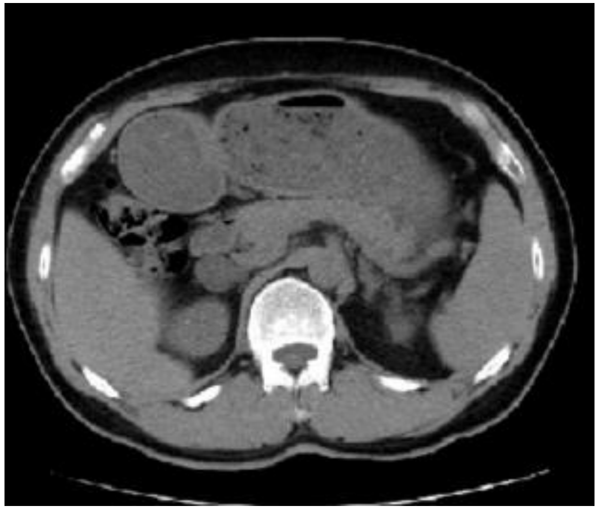	75	MACS	N	N	N	N	N	N	N

					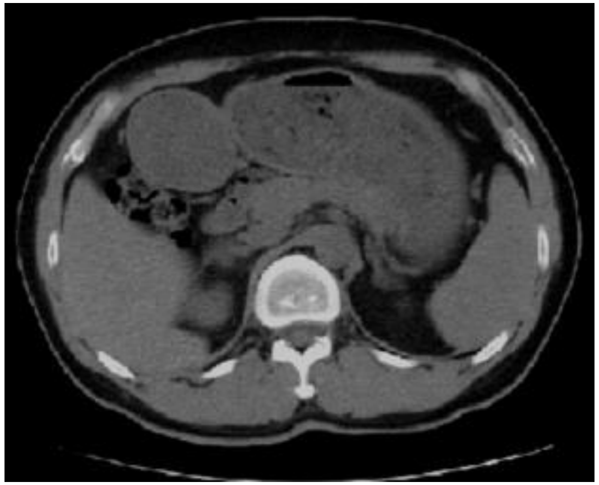									
P11	55	M	21.61	c.73del p.Glu25Argfs*16	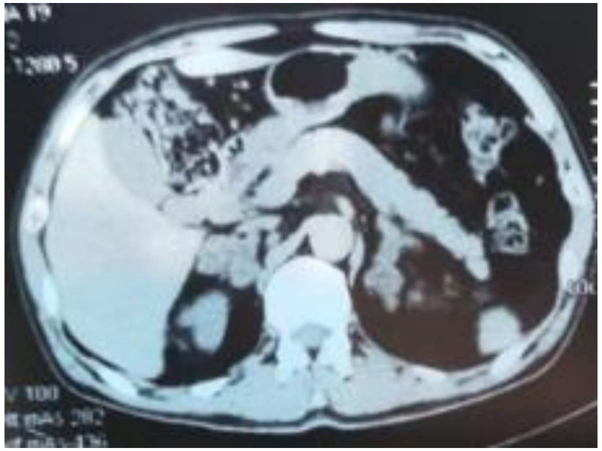	NA	MACS	Y	Y	N	Y	N	N	N

P12	61	F	28.52	c.294del<br>p.Gly99Glufs*38	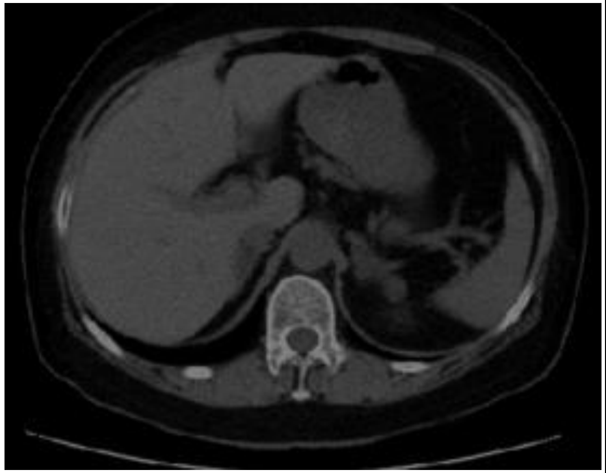<br>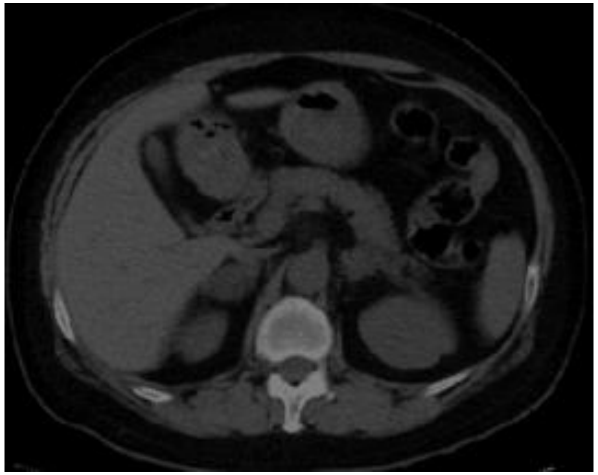	85	CS	Y	N	N	N	N	N	N

P13	47	F	25.11	c.1084C>T p.Arg362Trp	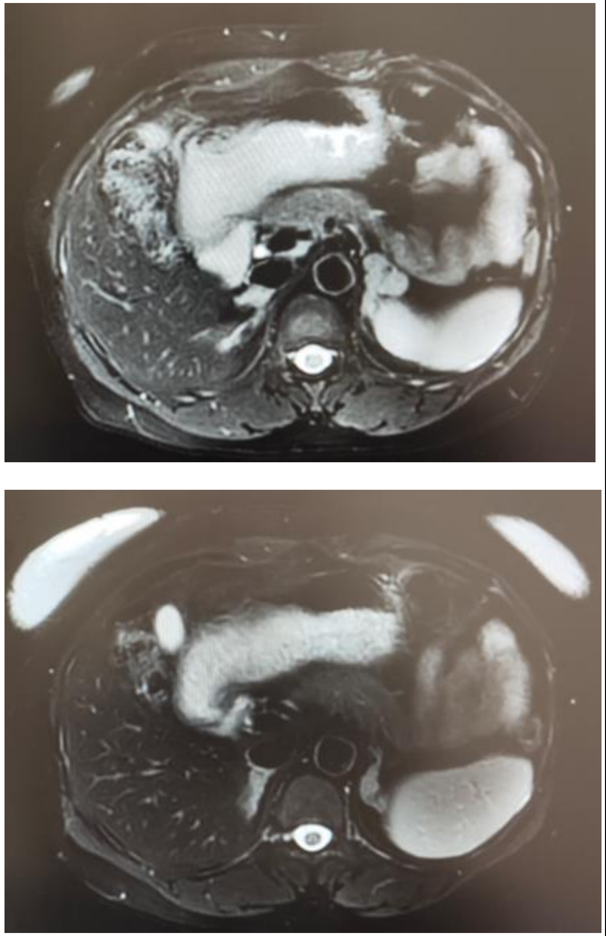	54	MACS	Y	N	N	N	N	N	N

P14	66	M	23.88	c.1084C>T p.Arg362Trp	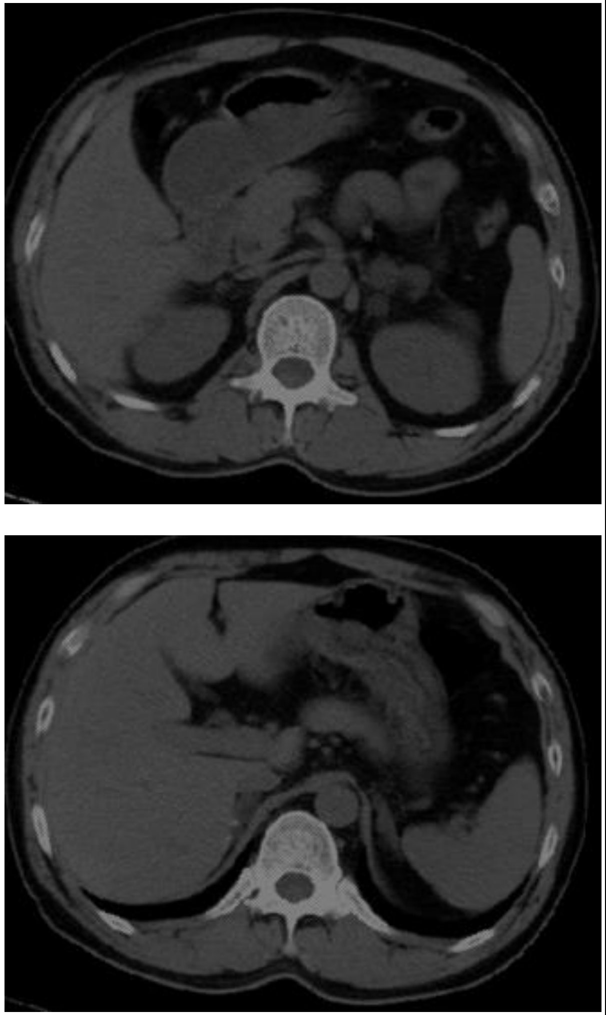	73	MACS	Y	N	Y	N	N	N	N

P15	67	M	24.96	c.256del<br>p.Gln86Argfs*51	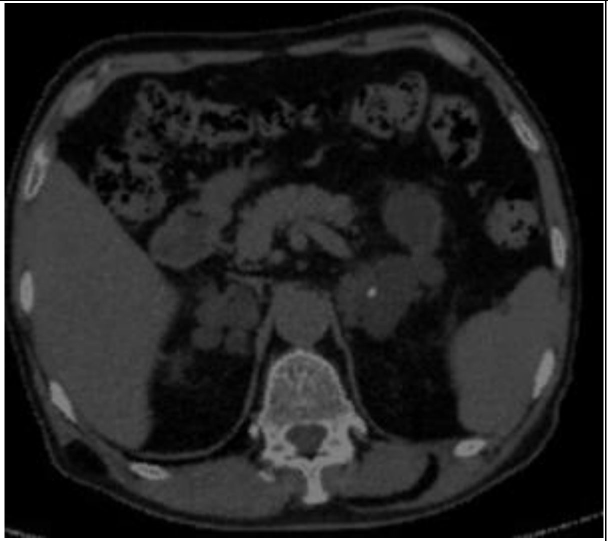	106	MACS	Y	Y	N	Y	N	Y	cerebral infarction
P16	50	M	22.84	c.2377C>T<br>p.Arg793*	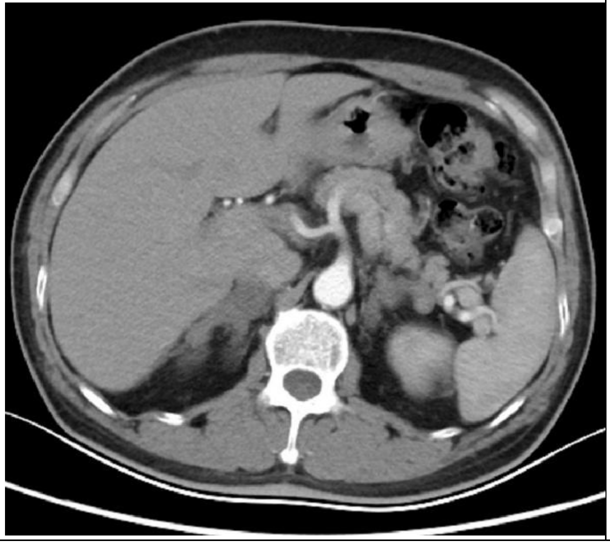	NA	MACS	Y	Y	Y	N	N	N	N

Supplementary Table 4. Literature review on adrenal imaging in patients with known ARMC5 genotype.

Abbreviations: WT, wild-type; MUT,mutated-type;Y, yes: N, no; NA, not available; CS, overt Cushing syndrome; MACS, mild autonomous cortisol secretion ; DST, dexamethasone suppression test.

Reference	DOI	Role	*ARMC5*	Figure numbers from the source	Key descriptions	Multiple confluent adrenal nodules	Endocrine type	Does it support that patients with *ARMC5* mutations present with multiple confluent adrenal nodules?
Khosla 2024(1)	10.1210/jcemcr/luad138	Sporadic	MUT(p.Cys829Arg)	Figure 1	CT: Massively enlarged bilateral adrenal glands	Y	MACS	Y
Hella 2023(2)	10.1556/650.2023.32817	Proband	MUT(c.1724del28 bp)	Figure 1	CT: Numerous nodules on each adrenal	Y	CS	Y
Tang 2023(3)	10.1186/s12902-023-01324-3	Sporadic	MUT(p. R619*)	Figure 1. A&B	CT: Bilateral adrenal masses	Y	CS	Y

					(right: 7.2 × 5.2 × 5.1 cm, left: 6.9 × 4.5 × 3.2 cm).			
Bertherat 2023(4)	10.1210/endrev/bnac034	NA	MUT	Figure 1. A,B&C	Unenhanced adrenal CT (A), enhanced adrenal CT (B) and T1-weighted adrenal MRI (C): several bilateral adrenal nodules, with an asymmetrical adrenal enlargement.	Y	NA	Y
Bouys 2022(5)	10.1530/EJE-21-1032	Unrelated	MUT	Figure 1.A	CT: Numerous nodules on each adrenal.	Y	NA	Y
		Unrelated	WT	Figure 1.B	CT: Unique nodule on each adrenal	N	NA	Y
Wang 2022(6)	10.1186/s12902-022-01128-x	Proband	MUT(p.Pro122Alafs*61)	Figure 1. a, b& c	CT: Bilateral adrenal glands			

					increased in size, with multiple nodular low-density shadows irregularly protruding, causing bilateral adrenal glands to appear "ginger-like" and enhanced with mildly uneven enhancement.			
Eghbali 2022(7)	10.3390/diagnostics12123028	Proband	MUT(p. Ala702Glu)	Figure 3.A&B	CT: Bilateral irregular adrenal masses, adrenal glands are replaced by multiple nodules	Y	CS+PA	Y
Vena 2022(8)	10.3389/fgene.2022.834067	Unrelated	MUT(germline:p.C813VFS*104;somatic: p.A77Afs*13)	Figure 1	CT: Bilateral enlargement of adrenal glands with a typical macro-nodular aspect of left	Y	MACS	Y

					adrenal.			
Cavalcante 2022(9)	10.1038/s41574-022-00718-y		MUT	Figure 1	CT: Macronodular adrenal glands.	Y	CS	Y
He 2021(10)	10.1186/s12920-021-00896-0	Proband	MUT(p. Gln323Ter)	Figure1.a	CT: Bilateral irregular adrenal masses.	Y	CS	Y
Ferreira 2020(11)	10.1155/2020/8848151	Proband	MUT(germline somatic:p.Leu460Pro)	Figure 1	CT: Bilateral adrenal incidentalomas	Y	CS	Y
Jojima 2020(12)	10.1530/EJE-20-0014	Proband	MUT(germline: p.R267*)	Figure 1.C	CT: Bilateral macronodular adrenal hyperplasia.	Y	MACS	Y
Wurth 2020(13)	10.1530/EDM-20-0006	Unrelated	MUT(p.R173*)	Figure 1.A	CT: Macronodular adrenocortical hyperplasia.	Y	CS	Y
		Unrelated	MUT(p.A362T)	Figure 1.B	CT: Macronodular adrenocortical hyperplasia.	Y	CS	Y
		Unrelated	WT	Figure 1.C	CT: Unique nodule on each adrenal.	N	CS	Y
Zhang	10.1507/endocrj.EJ20-0163	Proband	MUT(p.Gln 18X)	Figure 2	CT: Bilateral	Y	MACS	Y

2020(14)					adrenal enlargement, irregular morphology, and nodules.			
Mariani 2020(15)	10.3389/fendo.2020.00036	Proband	MUT(p.Arg362Trp)	Figure 1.A	CT: Volumetric increase and diffuse nodular thickening of both adrenal glands.	Y	MACS	Y
		Family member	MUT(p.Arg362Trp)	Figure 1.B	CT: Index-case’s sibling: bilateral enlargement of both adrenal glands with multiple hypoattenuating nodules.	Y	MACS	Y
		Family member	MUT(p.Arg362Trp)	Figure 1.C	CT: Index-case’s niece: normal without identifiable focal lesions.	N	Normal	N
		Unrelate	WT	Figure 2.A	CT: Bilateral	Y	NA	Y

		d			adrenal nodules.			
		Unrelated	WT	Figure 2.B	CT: Unilateral adrenal nodule.	N	NA	Y
		Unrelated	MUT(p.Arg654*)	Figure 3.A, B & C	Pre-contrast CT (A), post-contrast abdominal CT (B) and 18F-FDG-PET/CT (C): Bilateral adrenal nodules.	Y	CS	Y
Kyo 2019(16)	10.1210/js.2019-00210	Family member	MUT(p. R619*)	Figure 1.(b)	CT: A small adrenal nodule on the left adrenal gland (III-2).	N	Normal	N
		Family member	MUT(p. R619*)	Figure 1.(e)	CT: Left adrenal nodule (III-1)	N	Normal	N
		Family member	MUT(p. R619*)	Figure 1. (f) & (g)	CT: Normal adrenal glands (III-3).	N	Normal	N
Berthon 2019(17)	10.1016/j.jsbmb.2019.02.011	Sporadic	MUT(p.R 173*)	Figure 1	CT: Bilateral adrenal hyperplasia and internodular atrophy	Y	CS	Y
Yu 2018(18)	10.1371/journal.pone.0191602	Proband	MUT(p.Arg619*)	Supplemental figure 7.A	CT: Multiple adrenal nodules	Y	CS	Y

					(F1-II-5)			
		Family member	WT	Supplemental figure 7.B	CT: Only one nodule on the right side (F1-II-4).	N	CS	Y
		Family member	MUT(p.Arg619*)	Figure 1.D	CT: L: multiple nodules; R:multiple nodules (F1-II-1).	NA	CS	NA
		Sporadic	MUT(germline mutation)	Supplemental figure 5	CT: Multiple adrenal nodules.	Y	CS & PA	Y
		Family member	MUT(p.Arg764)	Figure 2.D	CT: L: multiple nodules; R: multiple nodules (F2-II-3).	NA	CS	NA
Liu 2018(19)	10.1186/s12881-018-0564-2	Sporadic	MUT(p.Arg173*)	Figure 1.a&b	CT: Bilateral adrenal masses.	Y	CS	Y
Jin 2018(20)	10.1186/s13256-017-1529-3	Sporadic	MUT(p.Gln228*)	Figure 1.a&b	Massive enlargement of the adrenal glands and multiple nodules	Y	CS	Y
Rego 2017(21)	10.1530/EDM-16-0135	Proband	MUT(germline: p.I58Nfs*45)	Figure 4	MRI: Bilateral enlarged adrenal glands, with lobulated	Y	CS	Y

					contours, heterogeneous nodular structure.			
		Family member	MUT(germline: p.I58Nfs*45)	Figure 2	CT: Bilateral enlarged adrenal glands, with lobulated contours (proband's daughter).	Y	severe hypercortisolism	Y
Albiger 2017(22)	10.1007/s12020-016-0956-z	NA	WT	Supplementary figure 1.a	CT: Bilateral hyperplastic adrenal enlargement (# 11)	N	CS	Y
		NA	MUT(p.Gly65Alafs72*)	Supplementary figure 1.b	CT: Larger, multinodular adrenal glands (# 24).	Y	CS	Y
Bourdeau 2016(23)	10.1530/EJE-15-0642	Family member	MUT(c.327_328insC)	Figure 3	CT: Bilateral enlargement of their adrenal glands with multiple nodules (II-3).	Y	MACS	Y
		Family	MUT(c.327_328insC)	Figure 3	CT: Bilateral	Y	CS	Y

		member			enlargement of their adrenal glands with multiple nodules (III-4).			
		Proband	MUT(c.327_328insC)	Figure 3	CT: Bilateral enlargement of their adrenal glands with multiple nodules (II-1).	Y	CS	Y
Espiard 2015(24)	10.1210/jc.2014-4204	Unrelated	MUT(p.A702_S706del)	Figure 3.A	CT: Massive enlargement of their adrenal glands and multiple nodules (patient 8) C and D, Wild-type patients have smaller adrenal hyperplasia (C, patient 97; D, patient 74).	Y	CS	Y
		Unrelated	MUT(p.A702_S706del)	Figure 3.B	CT: Massive enlargement of their adrenal	Y	CS	Y

					glands and multiple nodules (patient 9)			
		Unrelated	WT	Figure 3.C	CT: Unique nodule on each adrenal (patient 97).	N	CS	Y
		Unrelated	WT	Figure 3.D	CT: Unique nodule on each adrenal (patient 74).	N	MACS	Y
Elbelt 2015(25)	10.1210/jc.2014-2648	Proband	MUT (p.A110fs*9)	Figure 1. A&B	CT: Macronodular hyperplasia of the right (A) and left adrenal (B) (F1 VII).	Y	CS	Y
Suzuki 2015(26)	10.4158/EP15756.OR	NA	MUT(germline deletions in exons 1 to 5 of the ARMC5 gene locus)	Figure 2.A	MRI&CT: Enlargement of bilateral adrenal glands, with multiple nodules (mother).	Y	MACS	Y
		NA	MUT(germline deletions in exons 1 to 5 of the ARMC5 gene locus)	Figure 2.A	MRI&CT: Enlargement of bilateral adrenal	Y	MACS	Y

					glands, with multiple nodules (son).			
Correa 2015(27)	10.1530/EJE-15-0205	Unrelated	MUT(p.Trp476*)	Figure 1. A& B	CT: Bilateral multiple lobular masses more than 1 cm each in diameter, without evidence of cysts or microcalcifications.	Y	CS	Y
Alencar 2014(28)	10.1210/jc.2013-4237	Proband	MUT(germline:p.Leu365Pro;somatic: p.Cys657Trp)	Supplementary figure	CT: Left adrenal: thickened with nodules; right adrenal: thickened with nodules (IV-5).	Y	CS	Y
		Family member	MUT(germline:p.Leu365Pro)	Supplementary figure	CT: L Ad: normal; R Ad: thickened (III-13).	N	abnormal DST	N
		Family member	MUT(germline:p.Leu365Pro)	Supplementary figure	CT: L Ad: normal; R Ad: normal (III-18).	N	normal DST	N
		Family member	MUT(germline:p.Leu365Pro)	Supplementary figure	CT: L Ad: thickened with	Y	abnormal DST	Y

					nodules; R Ad: thickened with nodules (III-20).			
		Family member	MUT(germline:p.Leu365Pro)	Supplementary figure	CT: L Ad: normal; R Ad: with a nodule (IV-7).	N	abnormal DST	N
		Family member	MUT(germline:p.Leu365Pro)	Supplementary figure	CT: L Ad: normal; R Ad: thickened with nodules (V-1).	N	abnormal DST	N
		Family member	MUT(germline:p.Leu365Pro)	Supplementary figure	CT: L Ad: normal; R Ad: normal (V-3).	N	normal DST	N
Assié 2013(29)	10.1056/NEJMoa1304603	NA	MUT(germline:p.R267X;somatic: LOH, c.456–475+5del28, p.C139R, p.R619X)	Figure 3	CT: Various nodules present on both adrenal glands (Patient 5).	Y	CS	Y

References

1. Khosla S, Alsarraf F, & Nylen ES. Pituitary Macroadenoma With Macronodular Adrenal Hyperplasia and Novel Armadillo Repeat-Containing Protein 5 (ARMC5) Mutation. *JCEM Case Reports* 2024 **2** luad138. (doi:10.1210/jcemcr/luad138)

2. Hella Z, Tőke J, Patócs A, Varga Z, Dabasi G, Kovács GL, & Tóth M. Macronodular adrenal hyperplasia causing Cushing's syndrome due to ARMC5 gene mutation. *Orvosi Hetilap* 2023 **164** 1271–1277. (doi:10.1556/650.2023.32817)

3. Tang P, Zhang J, Peng S, Yan X, Wang Y, Wang S, Zhang Y, Liu G, Xu J, Huang Y, Zhang D, Liu Q, Jiang J, & Lan W. Primary bilateral macronodular adrenocortical

hyperplasia (PBMAH) patient with ARMC5 mutations. *BMC endocrine disorders* 2023 **23** 77. (doi:10.1186/s12902-023-01324-3)

4. Bertherat J, Bourdeau I, Bouys L, Chasseloup F, Kamenický P, & Lacroix A. Clinical, Pathophysiologic, Genetic, and Therapeutic Progress in Primary Bilateral Macronodular Adrenal Hyperplasia. *Endocrine Reviews* 2023 **44** 567–628. (doi:10.1210/endrev/bnac034)

5. Bouys L, Vaczlavik A, Jouinot A, Vaduva P, Espiard S, Assié G, Libé R, Perlemoine K, Ragazzon B, Guignat L, Groussin L, Bricaire L, Cavalcante IP, Bonnet-Serrano F, Lefebvre H, Raffin-Sanson ML, Chevalier N, Touraine P, Jublanc C, Vatier C, Raverot G, Haissaguerre M, Maione L, Kroiss M, Fassnacht M, Christin-Maitre S, Pasmant E, Borson-Chazot F, Tabarin A, … Bertherat J. Identification of predictive criteria for pathogenic variants of primary bilateral macronodular adrenal hyperplasia (PBMAH) gene *ARMC5* in 352 unselected patients. *European Journal of Endocrinology* 2022 **187** 123–134. (doi:10.1530/EJE-21-1032)

6. Wang W & Wei F. A novel pathogenic variant of ARMC5 in a patient with primary bilateral macronodular adrenal hyperplasia: a case report. *BMC endocrine disorders* 2022 **22** 211. (doi:10.1186/s12902-022-01128-x)

7. Eghbali M, Cheraghi S, Samanian S, Rad I, Meghdadi J, Akbari H, & Honardoost M. A Novel ARMC5 Germline Variant in Primary Macronodular Adrenal Hyperplasia Using Whole-Exome Sequencing. *Diagnostics* 2022 **12** 3028. (doi:10.3390/diagnostics12123028)

8. Vena W, Morelli V, Carrabba M, Elli F, Fabio G, Muller I, Lucca C, Maffini MA, Lania AG, Mantovani G, & Arosio M. Case Report: A Novel ARMC5 Germline Mutation in a Patient with Primary Bilateral Macronodular Adrenal Hyperplasia and Hypogammaglobulinemia. *Frontiers in Genetics* 2022 **13** 834067. (doi:10.3389/fgene.2022.834067)

9. Cavalcante IP, Berthon A, Fragoso MC, Reincke M, Stratakis CA, Ragazzon B, & Bertherat J. Primary bilateral macronodular adrenal hyperplasia: definitely a genetic disease. *Nature Reviews Endocrinology* 2022 **18** 699–711. (doi:10.1038/s41574-022-00718-y)

10. He WT, Wang X, Song W, Song XD, Lu YJ, Lv YK, He T, Yu XF, & Hu SH. A novel nonsense mutation in ARMC5 causes primary bilateral macronodular adrenocortical hyperplasia. *BMC Medical Genomics* 2021 **14** 126. (doi:10.1186/s12920-021-00896-0)

11. Ferreira MJ, Pedro J, Salazar D, Costa C, Aragão Rodrigues J, Costa MM, Grangeia A, Castedo JL, & Carvalho D. ARMC5 Primary Bilateral Macronodular Adrenal Hyperplasia Associated with a Meningioma: A Family Report. *Case Reports in Endocrinology* 2020 **2020** 1–5. (doi:10.1155/2020/8848151)

12. Jojima T, Kogai T, Iijima T, Kato K, Sagara M, Kezuka A, Kase M, Sakurai S, Akimoto K, Sakumoto J, Namatame T, Ueki K, Hishinuma A, Kamai T, Usui I, & Aso Y. Genetic alteration of ARMC5 in a patient diagnosed with meningioma and primary macronodular adrenal hyperplasia: a case report. *European Journal of Endocrinology* 2020 **183** K7–K12. (doi:10.1530/EJE-20-0014)

13. Wurth R, Kamilaris C, Nilubol N, Sadowski SM, Berthon A, Quezado MM, Faucz FR, Stratakis CA, & Hannah-Shmouni F. Inhibin A as a tumor marker for primary bilateral macronodular adrenal hyperplasia. *Endocrinology, Diabetes & Metabolism Case Reports* 2020 **2020** . (doi:10.1530/EDM-20-0006)

14. Zhang F, Lin X, & Yu X. Primary macronodular adrenal hyperplasia (PMAH) can be generated by a new *ARMC5* germline variant (c.52C>T (p.Gln18X)). *Endocrine Journal* 2020 **67** 1179–1186. (doi:10.1507/endocrj.EJ20-0163)

15. Mariani BMDP, Nishi MY, Wanichi IQ, Brondani VB, Lacombe AMF, Charchar H, Pereira MAA, Srougi V, Tanno FY, Ceccato F, Regazzo D, Barbot M, Occhi G, Albiger NME, Vieira-Corrêa M, Kater CE, Scaroni C, Chambô JL, Zerbini MCN, Mendonca BB, Almeida MQ, & Fragoso MCBV. Allelic Variants of ARMC5 in Patients With Adrenal Incidentalomas and in Patients With Cushing's Syndrome Associated With Bilateral Adrenal Nodules. *Frontiers in Endocrinology* 2020 **11** 36. (doi:10.3389/fendo.2020.00036)

16. Kyo C, Usui T, Kosugi R, Torii M, Yonemoto T, Ogawa T, Kotani M, Tamura N, Yamamoto Y, Katabami T, Kurihara I, Saito K, Kanamoto N, Fukuoka H, Wada N, Murabe H, & Inoue T. ARMC5 Alterations in Primary Macronodular Adrenal Hyperplasia (PMAH) and the Clinical State of Variant Carriers. *Journal of the Endocrine Society* 2019 **3** 1837–1846. (doi:10.1210/js.2019-00210)

17. Berthon A, Hannah-Shmouni F, Maria AG, Faucz FR, & Stratakis CA. High expression of adrenal P450 aromatase (CYP19A1) in association with ARMC5-primary bilateral macronodular adrenocortical hyperplasia. *The Journal of Steroid Biochemistry and Molecular Biology* 2019 **191** 105316. (doi:10.1016/j.jsbmb.2019.02.011)

18. Yu L, Zhang J, Guo X, Chen X, He Z, & He Q. ARMC5 mutations in familial and sporadic primary bilateral macronodular adrenal hyperplasia. *PloS One* 2018 **13** e0191602. (doi:10.1371/journal.pone.0191602)

19. Liu Q, Tong D, Xu J, Yang X, Yi Y, Zhang D, Wang L, Zhang J, Zhang Y, Li Y, Chang L, Chen R, Guan Y, Yi X, & Jiang J. A novel germline ARMC5 mutation in a patient with bilateral macronodular adrenal hyperplasia: a case report. *BMC Medical Genetics* 2018 **19** 49. (doi:10.1186/s12881-018-0564-2)

20. Jin P, Janjua MU, Zhang Q, Dong C sheng, Yang Y, & Mo Z hui. Extensive ARMC5 genetic variance in primary bilateral macronodular adrenal hyperplasia that started with exophthalmos: a case report. *Journal of Medical Case Reports* 2018 **12** 13. (doi:10.1186/s13256-017-1529-3)

21. Rego T, Fonseca F, Espiard S, Perlemoine K, Bertherat J, & Agapito A. ARMC5 mutation in a Portuguese family with primary bilateral macronodular adrenal hyperplasia (PBMAH). *Endocrinology, Diabetes & Metabolism Case Reports* 2017 **2017** . (doi:10.1530/EDM-16-0135)

22. Albiger NM, Regazzo D, Rubin B, Ferrara AM, Rizzati S, Taschin E, Ceccato F, Arnaldi G, Pecori Giraldi F, Stigliano A, Cerquetti L, Grimaldi F, De Menis E, Boscaro M, Iacobone M, Occhi G, & Scaroni C. A multicenter experience on the prevalence of ARMC5 mutations in patients with primary bilateral macronodular adrenal hyperplasia: from genetic characterization to clinical phenotype. *Endocrine* 2017 **55** 959–968. (doi:10.1007/s12020-016-0956-z)

23. Bourdeau I, Oble S, Magne F, Lévesque I, Cáceres-Gorriti KY, Nolet S, Awadalla P, Tremblay J, Hamet P, Fragoso MCBV, & Lacroix A. ARMC5 mutations in a large French-Canadian family with cortisol-secreting β-adrenergic/vasopressin responsive bilateral macronodular adrenal hyperplasia. *European Journal of Endocrinology* 2016 **174** 85–96. (doi:10.1530/EJE-15-0642)

24. Espiard S, Drougat L, Libé R, Assié G, Perlemoine K, Guignat L, Barrande G, Brucker-Davis F, Doullay F, Lopez S, Sonnet E, Torremocha F, Pinsard D, Chabbert-Buffet N, Raffin-Sanson ML, Groussin L, Borson-Chazot F, Coste J, Bertagna X, Stratakis CA, Beuschlein F, Ragazzon B, & Bertherat J. ARMC5 Mutations in a Large Cohort of Primary Macronodular Adrenal Hyperplasia: Clinical and Functional Consequences. *The Journal of Clinical Endocrinology and Metabolism* 2015 **100** E926-935. (doi:10.1210/jc.2014-4204)

25. Elbelt U, Trovato A, Kloth M, Gentz E, Finke R, Spranger J, Galas D, Weber S, Wolf C, König K, Arlt W, Büttner R, May P, Allolio B, & Schneider JG. Molecular and Clinical Evidence for an *ARMC5* Tumor Syndrome: Concurrent Inactivating Germline and Somatic Mutations Are Associated With Both Primary Macronodular Adrenal Hyperplasia and Meningioma. *The Journal of Clinical Endocrinology & Metabolism* 2015 **100** E119–E128. (doi:2015010511094700331)

26. Suzuki S, Tatsuno I, Oohara E, Nakayama A, Komai E, Shiga A, Kono T, Takiguchi T, Higuchi S, Sakuma I, Nagano H, Hashimoto N, Mayama T, Koide H, Sasano H, Nakatani Y, Imamoto T, Ichikawa T, Yokote K, & Tanaka T. Germline Deletion of Armc5 In Familial Primary Macronodular Adrenal Hyperplasia. *Endocrine Practice* 2015 **21** 1152–1160. (doi:10.4158/EP15756.OR)

27. Correa R, Zilbermint M, Berthon A, Espiard S, Batsis M, Papadakis GZ, Xekouki P, Lodish MB, Bertherat J, Faucz FR, & Stratakis CA. The ARMC5 gene shows

extensive genetic variance in primary macronodular adrenocortical hyperplasia. *European Journal of Endocrinology* 2015 **173** 435–440. (doi:10.1530/EJE-15-0205)

28. Alencar GA, Lerario AM, Nishi MY, Mariani BMDP, Almeida MQ, Tremblay J, Hamet P, Bourdeau I, Zerbini MCN, Pereira MAA, Gomes GC, Rocha MDS, Chambo JL, Lacroix A, Mendonca BB, & Fragoso MCBV. *ARMC5* Mutations Are a Frequent Cause of Primary Macronodular Adrenal Hyperplasia. *The Journal of Clinical Endocrinology & Metabolism* 2014 **99** E1501–E1509. (doi:10.1210/jc.2013-4237)

29. Assié G, Libé R, Espiard S, Rizk-Rabin M, Guimier A, Luscap W, Barreau O, Lefèvre L, Sibony M, Guignat L, Rodriguez S, Perlemoine K, René-Corail F, Letourneur F, Trabulsi B, Poussier A, Chabbert-Buffet N, Borson-Chazot F, Groussin L, Bertagna X, Stratakis CA, Ragazzon B, & Bertherat J. *ARMC5* Mutations in Macronodular Adrenal Hyperplasia with Cushing's Syndrome. *New England Journal of Medicine* 2013 **369** 2105–2114. (doi:10.1056/NEJMoa1304603)
